# Analyzing the role of *ACE2*, *AR*, *MX1* and *TMPRSS2* genetic markers for COVID-19 severity

**DOI:** 10.1186/s40246-023-00496-2

**Published:** 2023-06-07

**Authors:** Silvia Martinez-Diz, Carmen Maria Morales-Álvarez, Yarmila Garcia-Iglesias, Juan Miguel Guerrero-González, Catalina Romero-Cachinero, Jose María González-Cabezuelo, Francisco Javier Fernandez-Rosado, Verónica Arenas-Rodríguez, Rocío Lopez-Cintas, Maria Jesús Alvarez-Cubero, Luis Javier Martinez-Gonzalez

**Affiliations:** 1 Preventive Medicine and Public Health Service, Hospital Universitario Clínico San Cecilio, Granada, Spain; 2 GENYO, Centre for Genomics and Oncological Research: Pfizer, University of Granada, Andalusian Regional Government, PTS Granada, Granada, Spain; 3 Department of Biochemistry, Molecular Biology III and Inmunology, Faculty of Medicine, University of Granada, Parque Tecnológico de La Salud, Av. de La Investigación, 11, 18016 Granada, Spain; 4 Family Medicine, Health Sanitary Center, Zaidin Sur, Granada, Spain; 5 Nursery Department, DUE Sanitary Center Almanjayar, Granada, Spain; 6 Research and Development Department, Meridiem Seeds, Almería, Spain; 7 LORGEN G.P., PT, Ciencias de la Salud - BIC, Granada, Spain; 8 Family Medicine, Health Sanitary Center, Gran Capitán, Granada, Spain; 9 Biosanitary Research Institute (Ibs. GRANADA), University of Granada, Granada, Spain

**Keywords:** *ACE2*, Biomarker, *MX1*, SARS-CoV-2, *TMPRSS2*

## Abstract

**Background:**

The use of molecular biomarkers for COVID-19 remains unconclusive. The application of a molecular biomarker in combination with clinical ones that could help classifying aggressive patients in first steps of the disease could help clinician and sanitary system a better management of the disease. Here we characterize the role of *ACE2, AR, MX1, ERG, ETV5* and *TMPRSS2* for trying a better classification of COVID-19 through knowledge of the disease mechanisms.

**Methods:**

A total of 329 blood samples were genotyped in *ACE2*, *MX1* and *TMPRSS2*. RNA analyses were also performed from 258 available samples using quantitative polymerase chain reaction for genes: *ERG, ETV5, AR, MX1, ACE2,* and *TMPRSS2.* Moreover, in silico analysis variant effect predictor, ClinVar, IPA, DAVID, GTEx, STRING and miRDB database was also performed. Clinical and demographic data were recruited from all participants following WHO classification criteria.

**Results:**

We confirm the use of ferritin (*p* < 0.001), D-dimer (*p* < 0.010), CRP (*p* < 0.001) and LDH (*p* < 0.001) as markers for distinguishing mild and severe cohorts. Expression studies showed that *MX1* and *AR* are significantly higher expressed in mild vs severe patients (*p* < 0.05). *ACE2* and *TMPRSS2* are involved in the same molecular process of membrane fusion (*p* = 4.4 × 10^–3^), acting as proteases (*p* = 0.047).

**Conclusions:**

In addition to the key role of *TMPSRSS2*, we reported for the first time that higher expression levels of *AR* are related with a decreased risk of severe COVID-19 disease in females. Moreover, functional analysis demonstrates that *ACE2, MX1* and *TMPRSS2* are relevant markers in this disease.

**Graphical abstract:**

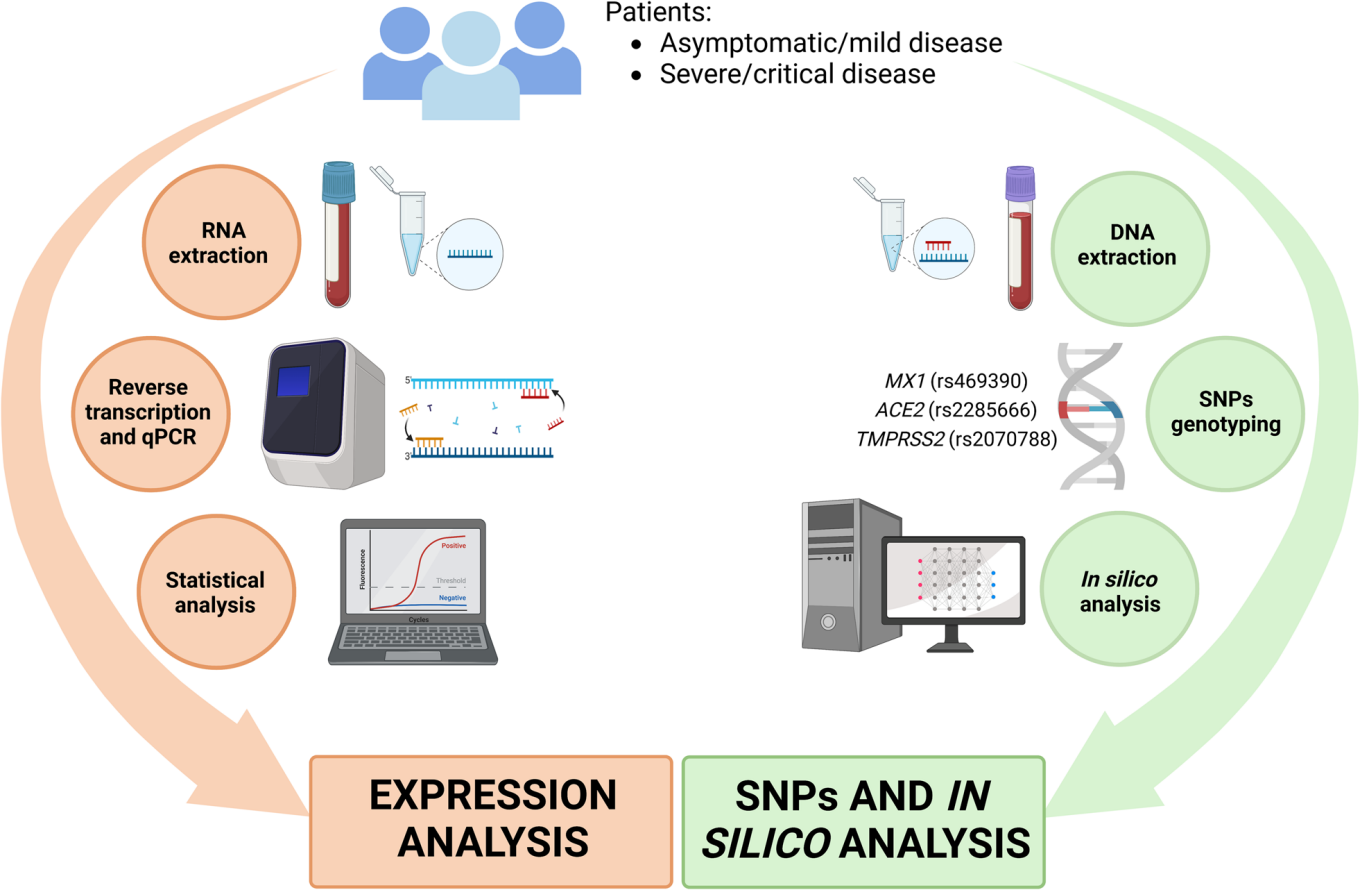

**Supplementary Information:**

The online version contains supplementary material available at 10.1186/s40246-023-00496-2.

## Introduction

Since the beginning of SARS-CoV-2, knowing the way of virus entrance in host cells is a challenge. Much emphasis has been placed in Transmembrane Serine Protease 2 (*TMPRSS2)* involved in the priming of viral S protein, mediating the cleavage of this viral spike [1]. Matsutama *et cols* demonstrated that *TMPRSS2* enhances SARS-CoV-2 infection by using cells expressing this receptor [2].

It is known that one of the main strategies to battle the virus is to avoid virus fusion in cell receptors, so we have focused on genes related to target receptor-binding domain of spike protein. One of the main genes is transmembrane serine protease 2 gene (*TMPRSS2*); which cleave the spike (S) protein. Moreover, there are studies suggesting that *TMPRSS2* rs75603675 genotype combined with dyslipidemia and gender, are main predictors of disease severity [3]. This gene acts jointly with *ACE2*, and both have been included as the two major host factors contributing to SARS-CoV-2 virulence and pathogenesis [4]. There are a growing number of studies showing the abnormal predominance of pro-inflammatory ACE/Ang II/AT1R/Nox over anti-inflammatory ACE2/Angiotensin/MasR pathways as the probable cause of chaotic inflammatory responses in COVID-19 [4]. The human myxovirus resistance genes (*MX*) encode GTPases that are part of the antiviral response induced by type I/III IFNs. *MX1* is proved with a wide antiviral activity against RNA and DNA viruses, and it partially overlaps with *TMPRSS2* transcript [5]. Moreover, it has been proved that five single nucleotide polymorphisms (SNPs) within *TMPRSS2* and near *MX1* gene show associations with severe COVID-19 [6]. Furthermore, recent publications have related methylation and expression altered levels of *MX1* in the critical group as indicators of the role of SARS-CoV-2 in reducing the expression levels of this antiviral gene and thus promoting virus replication and disease progression [7].

Furthermore, drugs designing for S*AR*S-CoV -2 was even proposed and approved by FDA target *TMPRSS2* and angiotensin converting enzyme 2 (*ACE2*), suggested as novel antiviral drugs [8]. These both enzymes (*TMPRSS2* and *ACE2*) are considered as main points of cell-mediated viral entry, and several alkaloids are also being evaluated as drug inhibitors for these enzymes, and as novel drug strategies in COVID-19 [9].

Moreover, in prostate cancer cells it has been described an effect of androgens on *AR* (androgen receptor) increasing *TMPRSS2* expression, suggesting the correlation of increased lung infections (and severe COVID-19) in men [10, 11]. Furthermore, it has been identified a co-expression of *ACE2*, *TMPRSS2*, and *AR* in human alveolar and bronchial epithelial cells, indicating that *AR* drives *ACE2* and *TMPRSS2* expression in the lung [12]. Likewise, recent data have suggested *AR* as anti-infective target for anti-Omicron lineages, mainly due to the specificity of *AR* against spike RBD (receptor binding protein) of S*AR*S-CoV -2 [13].

*TMPRSS2* is also strongly related with other genes involved in prostate cancer, like *ERG*, *ETV5* and *AR*, whose characterization could help to clearly classifying COVID-19 [7, 11]. *AR* is also reported as having a role in SARS-CoV-2 mediating fibrotic and immune responses in the lung, like collagen deposition and cytokine levels. *TMPRSS2* is a well-known transcriptional target of *AR* in prostate cancer cells and has recently shown to be regulated by androgens and anti-androgens also in lung cells and mouse lung tissue [12]. The role of *ERG* together with *TMPRSS2* in non-prostatic tissues remains to be elucidated, but it has been shown that this gene may be involved in inflammatory responses [14].

As can be seen, *TMPRSS2* is a gene that plays a pivotal role in COVID-19 development. *TMPRSS2* is demonstrated as an essential host cell factor in the context of SARS-CoV-2 infection and constitutes an attractive target for antiviral intervention [15]. However, the other genes mentioned above (apart from ACE* 2*), they still do not have strong evidence in COVID-19 disease, while we have demonstrated a robust link among all of them. Our main aim is to try a molecular characterization in these main related genes (*TMPRSS2, AR, ACE2* and *MX1*) to better understand COVID-19 severity; and trying to initiate the lines for molecular biomarkers in COVID-19 management.

## Results

### Clinical analysis

We have performed an analysis comparing clinical data in present cohort (asymptomatic vs severe disease). We found that age has significant values (*p* < 0.001), severe patients ranged around 60.1 years old contrasting to mild ones around 45.3. Moreover, clinical parameters such as increased ferritin (*p* < 0.001), D-dimer (*p* < 0.010), C-Reactive protein (CRP) (*p* < 0.001) and lactate deshidrogenase (LDH) (*p* < 0.001) present significant values. All of them, except D-dimer, present the maximum proportion of the population with higher values in severe groups of patients. But D-dimer has a 60.7% of mild disease patients located with the highest values (> 4.77 ng/mL); more details of clinical data are described in Additional file 1: Table S1. Details in Table 1.

**Table 1:** Characteristics of the study population

	Asymptomatic/mild disease n (%)	Severe/critical disease n (%)	OR (CI 95%)	*p*-value*
Age (n = 330)	45.3 ± 15.3**	60.1 ± 12.8**		** < 0.001**
< 55 years	126 (67.4%)	59 (41.3%)	Ref.	** < 0.001**
≥ 55 years	61 (32.6%)	84 (58.7%)	2.94 (1.87–4.62)	
Gender (n = 330)				0.477
Male	85 (45.7%)	71 (49.7%)	Ref.	
Female	102 (54.3%)	72 (50.3%)	0.86 (0.55–1.33)	
Clinical data				
Ferritin (ng/mL) (n = 216)				** < 0.001**
< 258	73 (62.4%)	32 (32.3%)	Ref.	
> 258	44 (37.6%)	67 (67.7%)	3.47 (1.97–6.10)	
D-dimer (ng/mL) (n = 198)				** < 0.010**
< 4.77	42 (39.9%)	53 (58.2%)	Ref.	
> 4.77	65 (60.7%)	38 (41.8%)	0.46 (0.26–0.82)	
CRP (mg/L) (n = 211)				** < 0.001**
< 19.8	74 (65.5%)	31 (31.6%)	Ref.	
> 19.8	39 (34.5%)	67 (68.4%)	4.10 (2.30–7.29)	
Troponin (ng/L) (n = 71)				0.440
< 9	11 (44%)	15 (32.6%)	Ref.	
> 9	14 (56%)	31 (67.4%)	1.62 (0.59–4.42)	
LDH (U/L) (n = 185)				** < 0.001**
< 262	61 (64.2%)	33 (36.7%)	Ref.	
> 262	34 (35.8%)	57 (63.3%)	3.09 (1.70–5.64)	
IL-6 (pg/mL) (n = 21)				0.670
< 51	4 (40%)	6 (54.5%)	Ref.	
> 51	6 (60%)	5 (45.5%)	0.56 (0.09–3.14)	
Influenza vaccine (n = 198)				** < 0.001**
No	90 (70.3%)	32 (45.7%)	Ref.	
Yes	38 (29.7%)	38 (54.3%)	2.82 (1.53–5.14)	
ICU clinical follow-up				
Artificial respiration (n = 88)				0.991
No	8 (30.8%)	19 (30.6%)	Ref.	
Yes	18 (69.2%)	43 (69.4%)	1.00 (0.37–2.71)	
Pneumonia (n = 330)				
No	155 (82.9%)	80 (55.9%)	Ref.	** < 0.001**
Yes	32 (17.1%)	63 (44.1%)	3.81 (2.30–6.31)	
Systemic inflammatory r. (n = 43)				** < 0.001**
No	4 (33.3%)	11(35.5%)	Ref.	
Yes	8 (66.6%)	20 (64.5%)	4.29 (1.33–13.82)	
Days hospitalized (n = 81)				** < 0.001**
< 37 days	21 (87.5%)	47 (83.9%)	Ref.	
≥ 37 days	4 (12.5%)	9 (16.1%)	4.16 (2.34–7.41)	
Symptomatology				
Skin affection (n = 131)				0.077
No	62 (96.9%)	59 (88.1%)	Ref.	
Yes	2 (3.1%)	8 (11.9%)	4.20 (0.85–20.61)	
Anosmia (n = 143)				0.800
No	59 (81.9%)	57 (80.3%)	Ref.	
Yes	13 (18.1%)	14 (19.7%)	1.11 (0.48–2.57)	
Ageusia (n = 144)				1.000
No	56 (77.8%)	56 (77.8%)	Ref.	
Yes	16 (22.2%)	16 (22.2%)	1 (0.45–2.19)	
Myalgia (n = 140)				0.270
No	44 (64.7%)	40 (55.6%)	Ref.	
Yes	24 (35.3%)	32 (44.4%)	1.46 (0.74–2.89)	
Headache (n = 140)				0.225
No	53 (74.6%)	45 (65.2%)	Ref.	
Yes	18 (25.4%)	24 (34.8%)	1.57 (0.75–3.25)	
Fever (n = 172)				**0.033**
No	42 (49.4%)	29 (33.3%)	Ref.	
Yes	43 (50.6%)	58 (66.7%)	1.95 (1.05–3.62)	
Dyspnoea (n = 143)				0.360
No	41 (56.9%)	35 (49.3%)	Ref.	
Yes	31 (43.1%)	36 (50.7%)	1.36 (0.70–2.62)	
Asthenia (n = 130)				0.722
No	33 (50%)	30 (46.9%)	Ref.	
Yes	33 (50%)	34 (53.1%)	1.13 (0.56–2.26)	
Long Covid (n = 69)				0.385
No	26 (61.9%)	14 (50%)	Ref.	
Yes	15 (35.7%)	14(50%)	1.62 (0.62–4.27)	
Comorbidities				
Cancer (n = 3)	1(100%)	3 (10%)		
Obesity (n = 18)	0 (0%)	18 (90%)		

*Chi square (except Student t-test for age)

**Mean ± Standard Deviation

In bold statistically significant values

### Genetic analysis

We assessed TaqMan® genotyping analysis in three main selected SNPs of genes *ACE2* (rs2285666), *MX1* (rs469390) and *TMPRSS2* (rs2070788) for present analysis. As it will be described in detail in “Genotyping” section Genotyping; the selection was performed according to their relevance in NCBI (COVID-19 issue) and their allelic frequency (over 10% in minor allele in Caucasian population). We have conducted “Genetic analysis” section in two phases; (1) How genetic markers could stratify COVID-19 aggressiveness (classifying the disease in asymptomatic/mild vs Severe/critical; and (2) How clinical parameters (ferritin, D-dimer, CRP, troponin, lactate dehydrogenase and IL-6) correlate to genetic markers. Firstly, we have conducted how genetic markers could help to stratify our cohort (mild vs severe), but none of them had significant values. We found that G carriers in *TMPRSS2* (rs2070788) have more risk of developing serious/critical disease, especially in women (Table 2).

**Table 2:** Logistic regression analysis of risk of COVID-19 disease

	Asymptomatic/mild disease N (%)	Severe/critical disease N (%)	OR (CI 95%)	*p*-value*
*MX1*				0.575
Male				
Codominant				
AA	23 (20.1%)	21 (29.6%)		0.491
AG	47 (55.3%)	33 (46.5%)		
GG	15 (17.6%)	17 (23.9)		
Dominant				
AA	23 (20.1%)	21 (29.6%)	Ref.	0.728
AG + GG	62 (72.9%)	50 (70.4%)	0.883 (0.43–1.77)	
Recessive				
GG	15 (17.6%)	17 (23.9%)	Ref.	0.332
AA + AG	70 (82.4%)	54 (76.1%)	0.68 (0.32–1.49)	
Female				
Codominant				
AA	26 (37.7%)	26 (18.4%)		0.269
AG	52 (53.1%)	33(47.8%)		
GG	20 (20.4%)	10 (14.5%)		
Dominant				
AA	26 (26.5%)	26 (37.7%)	Ref.	0.125
AG + GG	72 (73.5%)	43 (62.3%)	0.59 (0.31–1.15)	
Recessive				
GG	20 (20.4%)	10 (14.5%)	Ref.	0.327
AA + AG	78 (79.6%)	59 (85.5%)	1.51 (0.66–3.47)	
*ACE2*				0.855
Male**				
C allele	69 (81.2%)	56 (78.9%)	Ref.	0.720
T allele	16 (18.8%)	15 (21.1%)	1.15 (0.53–2.54)	
Female				
Codominant				
CC	65 (66.3%)	45 (65.2%)		0.667
CT	32 (32.7%)	22 (31.9%)		
TT	1 (1%)	2 (2.9%)		
Dominant				
TT	1 (1%)	2 (2.9%)	Ref.	0.368
CT + CC	97 (99%)	67 (97.1%)	0.34 (0.03–3.88)	
Recessive				
CC	65 (66.3%)	45 (65.2%)	Ref.	0.882
CT + TT	33 (33.7%)	24 (34.8%)	1.05 (0.55–2.01)	
*TMPRSS2*				0.460
Male				
Codominant				
AA	28 (33.3%)	25 (35.2%)		0.967
AG	41 (48.5%)	34 (47.9%)		
GG	15 (17.9%)	12 (16.9%)		
Dominant				
AA	28 (33.3%)	25 (35.2%)	Ref.	0.806
AG + GG	56 (66.7%)	46 (64.8%)	0.92 (0.47–1.79)	
Recessive				
GG	15 (17.9%)	12 (16.9%)	Ref.	0.876
AA + AG	69 (82.1%)	59 (83.1%)	1.07 (0.46–2.46)	
Female				
Codominant				
AA	41 (41.8%)	16 (23.2%)		0.036
AG	39 (39.8%)	39 (56.5%)		
GG	18 (18.8%)	14 (20.3%)		
Dominant				
AA	41 (41.8%)	16 (23.2%)	Ref.	**0.012**
AG + GG	57 (58.2%)	53 (76.8%)	2.38 (1.20–4.74)	
Recessive				
GG	18 (18.4%)	14 (20.3%)	Ref.	0.756
AA + AG	80 (81.6%)	55 (79.7%)	0.88 (0.41–1.93)	

*Pearson's chi-squared test (χ^2^)

**ACE2 rs2285666 is located in X chromosome, so we only found C allele or T allele in male cases

In bold statistically significant values

Secondly, we have focused on the analysis of these SNPs in relation to main clinical parameters such as ferritin, D-dimer, CRP, troponin, lactate dehydrogenase and interleukin-6 (IL-6), but no significant values were shown. See Additional file 1: Tables S2 and S3 for more details. A meta-analysis was also performed but not relevant data were obtained, see details in Additional file 1: Table S9.

### Allele combination analysis

SNPs association studies could be a better predictive approach rather than investigating individual polymorphisms. It estimates more specific risk, reduces the dimension of association test, and increase statistical power.

The most common allele combination for *ACE2, MX1* and *TMPRSS2* in present study was TAG with frequencies of 24.59% in mild patients and 24.74% in severe patients (Additional file 1: Table S4). None of the combinations were significantly associated with a reduced risk of severe COVID-19, however we observed that TAA (OR 0.25, 95% CI 0.06–1.09;* p* = 0.066) could work as a protective role. SNPs combination CAA (OR 6.27; 95% CI 1.00–39.22; *p* = 0.051), was significantly associated with an increased risk of developing severe COVID-19.

### Gene expression analysis

**Fig. 1:**
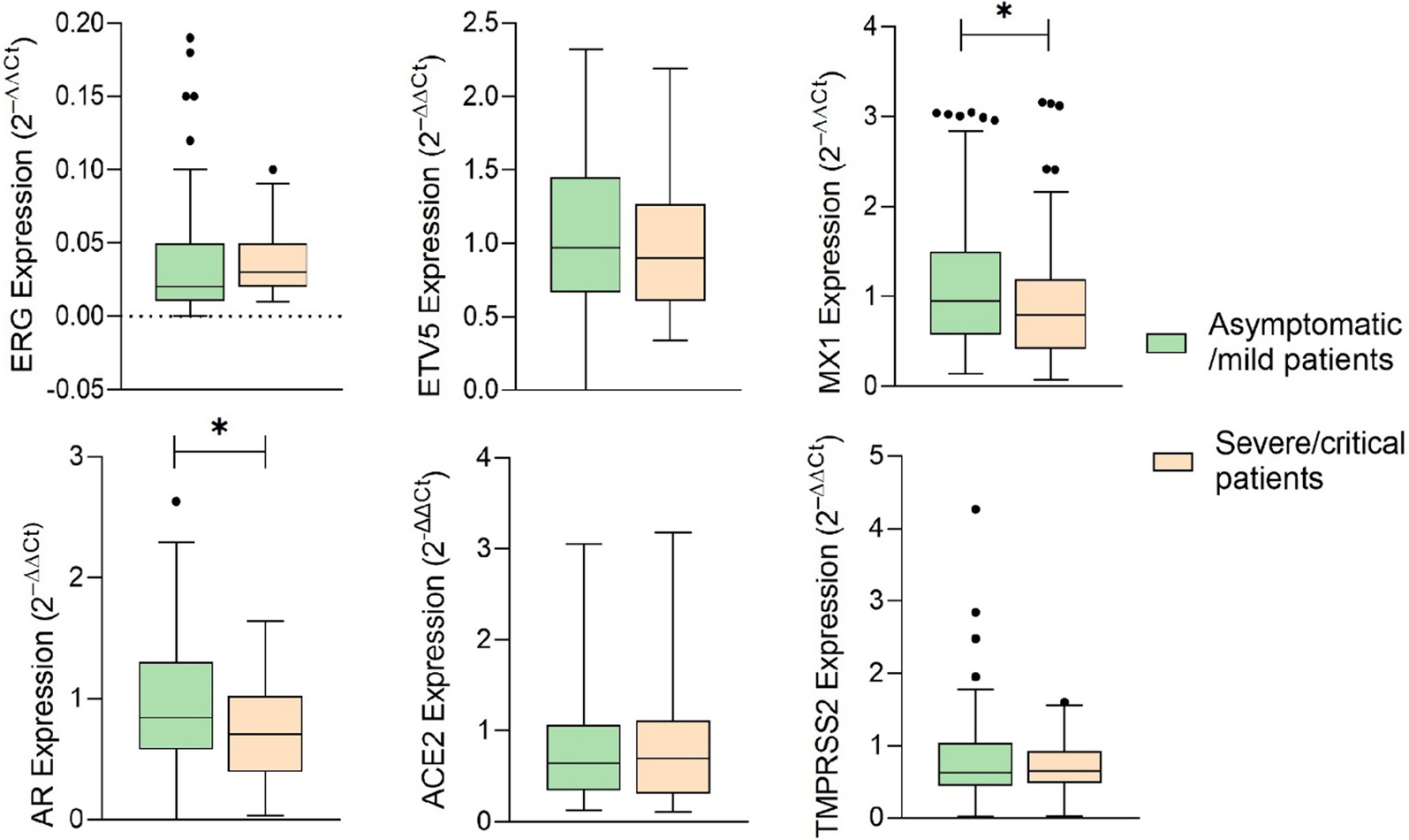
Genes expression analysis comparing aggressiveness in COVID-19 disease. (*)* p* < 0.05

Differential expression analysis was performed as previously described. The value of genetic expression of each patient was calculated as the average ± SD (standard deviation) of three different replicates. A Tukey’s range test was performed to detect anomalous values. As can be seen in Additional file 1: Table S5, when comparing aggressiveness, *AR* and *MX1* had statistically significant differences (*p* = 0.002 and *p* = 0.036 respectively), showing higher expression in asymptomatic/mild patients (Fig. 1).

The association between sociodemographic factors (age and gender) with gene expression levels of COVID-19 aggressiveness is shown in Table 3. Younger age and higher expression levels in *ERG* increased the risk of severe COVID-19 (*p* = 0.042). In the case of *AR* higher expression levels are related with a decreased risk of severe COVID-19 disease (*p* = 0.025) in female cases.

**Table 3:** Binary logistic regression model for the aggressiveness in COVID-19 disease assessed by age and gender

Variable	Gene	Expression level	Asymptomatic/mild disease; N	Severe/critical disease; N	*p*-value	OR (CI 95%)
< 55 years old	*ERG*	Low	58	10	**0.042**	Ref.
		High	48	20		2.471 (1.033–5.654)
	*ETV5*	Low	60	18	0.606	Ref.
		High	69	17		0.821 (0.389–1.735)
	*AR*	Low	56	12	0.473	Ref.
		High	66	19		1.343 (0.600–3.007)
	*MX1*	Low	56	17	0.276	Ref.
		High	67	13		0.639 (0.286–1.429)
	*ACE2*	Low	55	13	0.973	Ref.
		High	50	12		1.015 (0.424–2.431)
	*TMPRSS2*	Low	21	5	0.512	Ref.
		High	19	7		1.547 (0.420–5.704)
≥ 55 years old	*ERG*	Low	9	16	0.983	Ref.
		High	10	18		1.012 (0.329–3.117)
	*ETV5*	Low	10	22	0.625	Ref.
		High	9	15		0.758 (0.249–2.309)
	*AR*	Low	8	28	0.016	Ref.
		High	11	9		0.234 (0.072–0.761)
	*MX1*	Low	12	20	0.907	Ref.
		High	9	16		1.067 (0.360–3.159)
	*ACE2*	Low	10	11	0.560	Ref.
		High	11	17		1.405 (0.448–4.410)
	*TMPRSS2*	Low	3	2	0.872	Ref.
		High	5	4		1.200 (0.130–11.052)
Male	*ERG*	Low	26	4	0.131	Ref.
		High	27	11		2.648 (0.748–9.380)
	*ETV5*	Low	23	7	0.795	Ref.
		High	38	10		0.865 (0.289–2.587)
	*AR*	Low	31	7	0.414	Ref.
		High	25	9		1.594 (0.520–4.884)
	*MX1*	Low	34	7	0.698	Ref.
		High	27	7		1.259 (0.394–4.029)
	*ACE2*	Low	35	6	0.156	Ref.
		High	8	4		2.917 (0.664–12.813)
	*TMPRSS2*	Low	7	2	0.796	Ref.
		High	8	3		1.312 (0.168–10.264)
Female	*ERG*	Low	41	22	0.167	Ref.
		High	27	11		1.677 (0.805–3.494)
	*ETV5*	Low	46	33	0.448	Ref.
		High	40	22		0.767 (0.386–1.552)
	*AR*	Low	32	33	**0.005**	Ref.
		High	52	19		0.354 (0.173–0.725)
	*MX1*	Low	33	30	0.140	Ref.
		High	49	22		0.471 (0.173–1.280)
	*ACE2*	Low	29	18	0.477	Ref.
		High	53	25		0.760 (0.357–1.619)
	*TMPRSS2*	Low	17	5	0.427	Ref.
		High	16	8		1.700 (0.549–6.297)

In bold those statistically significant values

### In silico* analysis*

Most of the variant changes are in an intron or upstream/downstream genes, and they do not produce any amino acid change. See details in Additional file 1: Table S6. As expected, data from DAVID Bioinformatics Resources confirmed that present studied genes have a strong association with coronavirus disease, according to their clinical implication. *ACE2, MX1* and *TMPRSS2* were found in the disease development pathway (*p* = 8.1 × 10^–4^). *ACE2* and *TMPRSS2* are involved in early stages of infection, while *MX1* implication takes place in later stages. Moreover, two of the genes of interest (*ACE2* and *TMPRSS2*) are supposed to be very closely related and involved in the same molecular process of membrane fusion (*p* = 4.4 × 10^–3^), where both are acting as proteases (*p* = 0.047). Also, it is obtained that most of the studied genes (*TMPRSS2*, *ERG*, *ETV5* and *AR*) are associated with pathways involved in the development of prostate cancer. It is also possible to infer a relationship of these genes with the transduction of signals that induce membrane fusion (*p* = 0.011), and with a positive regulation of transcription from RNA polymerase II (*p* = 0.034).

Interestingly, GTEx data showed a significantly higher expression of *TMPRSS2* in lung tissue with GG genotype in rs2070788 (*p* = 8.9 × 10^–9^), and in *MX1* gene (rs469390) in AA (*p* = 9 × 10^–8^). Although we did not observe significant differences in our samples, our expression analysis were done in blood samples and showed a similar shift for rs469390, see details in Additional file 1: Fig. S1.

In silico analysis using different miRNAs target prediction tools showed numerous miRNAs potentially targeting *ACE2, MX1* and/or *TMPRSS2* genes. Among all miRNAs predicted, only a few were related to the respiratory system. Finally, eleven miRNAs were highlighted as master regulators of studied genes (Additional file 1: Fig. S2).

Moreover, STRING analysis also reported a close relationship of *TMPRSS2* with the remaining studied genes. It shares an implication in prostate cancer with *ETV5*, *AR* and *ERG*; while *ACE2* shares its importance in the development of COVID-19. On the other hand, the study of *MX1* is also important due to its physical proximity to *TMPRSS2* within chromosome 21, although they are not connected in these pathways (Additional file 1: Fig. S3).

## Discussion

Characterization of molecules involved in the infection process for classifying COVID-19 severity remains a challenge in present clinical practice. A deeper understanding of mechanisms for SARS-CoV-2 infection involves investigating the host proteins used by this virus, such as *TMPRSS2* [16–18].

Here we focus on *TMPRSS2*, and related genes *ACE2*, *MX1*, *AR*, *ETV5* and *ERG*, by its own or combined with clinical data, as an easy and relevant classifier of COVID-19 aggressiveness. Although there are many publications concerning this aim, there are controversial data about this goal.

A multi-omic approach developed by JS Maras et al., demonstrated that increased basal level of *MX1* is correlated with SARS-CoV-2 infection [19]. This event could aid in the identification of patients predisposed to high severity. Moreover, many efforts are focused on the role of *ACE2* and *TMPRSS2* as key markers for COVID-19 severity. It has been demonstrated that human endothelial cells express the main cofactors needed for SARS-CoV-2 internalization, including *ACE2*, *TMPRSS2*, and CD-147 [20]; so directly or indirectly they will be involved in the disease.

This is the first time that *MX1* gene (rs469390) is related to COVID-19 and proved its utility as expression biomarker between asymptomatic and severe patients. *MX1* is reported as a gene product of interferon, which will play important roles in inflammation in the lower respiratory tract, which will be relevant for developing severe COVID-19 cases. Moreover, *MX1* is included as a calculator of “inflammation index” highly expressed in COVID-19 patients with a high diagnostic yield [21] and suggested as a good respiratory biomarker due to its interaction with *TMPRSS2*. However, here according to severity or clinical parameters, we could not find differences in rs469390 of *MX1* gene, although by its interaction with *TMPRSS2* was suggested to be a good respiratory biomarker [22].

According to *ACE2*, it is recognized as the main receptor for SARS-CoV-2 and it is a requirement for COVID-19 virus entry combined with *TMPRSS2*. High expressions patterns of *ACE2* and Dipeptidyl Peptidase 4 (DPP4) can be detected in blood and alveolar lavage fluid in patients with chronic obstructive pulmonary disease and asthma [21, 23]. We selected its main SNP (rs2285666), but no significant values were reported. Although it was previously described in a Caucasian population associated with an increased risk of being hospitalized and a severity course of the disease with recessive models of inheritance [22], the same was reported in Indian populations [24]. This SNP is in intron 3, and it was also reported that intronic regions play relevant regulation in *ACE2*. Specifically, it has been suggested that a reduced expression of *ACE2* may lead to an imbalance of the renin-angiotensin system in patients with COVID-19, which may represent a major pathological outcome of viral infection [25]. A study developed in Iranian population indicated that rs2285666 GG genotype or G allele by its own is associated with the incidence of COVID-19 [26].

In relation to *TMPRSS2*, we focused on rs2070788 which was suggested with interest on virus infections, in combination with other SNPs in present gene. Similarly, as described by K. Schonfelder et al. in a German cohort, we could not find a relation between this SNP and infection risk or severity in COVID-19 [1]. Moreover, it has also been reported that rs2070788 with GG genotype had the highest expression in lung compared to other genotypes [22]. Accordingly, here we found that G carriers in *TMPRSS2* (rs2070788) have more risk of developing serious/critical disease, especially in women. Furthermore, GTEx data showed a higher expression of GG genotype in lung tissue, in contrast to our expression analyses that showed lower expression level of this gene in G carriers patients. This might be caused by variations in expression between the different tissue types. This association between expression and disease severity could be due to an increased easiness of this protein to be found by the virus in the cellular membrane, resulting in a higher infection success [5, 6]. It was also found a relationship between rs469390 in *MX1* and a higher expression level in lung of A carriers in GTEx database. These data are in accordance with our results and previous observations of AA genotype association and a higher susceptibility to COVID-19.

*TMPRSS2* is a protein belonging to the serine protease family, which functions rely on gene fusion with ETS transcription factors, such as *ERG* and ETV1. The *TMPRSS2*: *ERG* gene fusion is the most frequent genomic alteration in several tumour cases and results in overexpression of the transcription factor *ERG* [20]. Other authors suggested that COVID-19 severity is higher in men, and this is due to an important role of androgens in SARS-CoV-2 infection mechanism [7, 10, 11]. That is why we have also included *ERG* and *AR* expression analysis in present study. We found that higher expression levels in *ERG* increased the risk of severe COVID-19 which is described for the first time with this role. It has been described as a potential drug target for treatment of COVID-19, but nothing is reported as a severity biomarker [27].

Moreover, we also found that *AR* expression is altered between asymptomatic/mild and severe/critical patients. This discovery is in the same line of reported data in LNCaP cancer cells, those treated with *AR* antagonists of prostate cancer (apalutamide, darolutamide, and enzalutamide) have an inhibition of S*AR*S-

CoV-2 infection [7]. Furthermore, disparities in gender infection of COVID-19 are suggested due to higher levels of *ACE2* and *TMPRSS2* in males, as well as hormonal influences on the immune response [7].

Moreover, when developing VEP and DAVID analysis, it reinforces the strong association with coronavirus disease, according to their clinical implication; these three genes and their variants. STRING analysis corroborated these results, emphasizing the role of *ACE2* and *TMPRSS2* as interesting biomarkers of COVID-19.

Furthermore, we also found that *ACE2*, *MX1* and *TMPRSS2* TAA could work as a protective role contrasting with CAA significantly associated with an increased risk of developing severe COVID-19. This is the first time that a combination of these SNPs is performed to associate with COVID-19 risk.

Recent studies are focusing on the search of miRNAs that target main genes related to COVID-19 aggressiveness like *ACE2* and *TMPRSS2*. These analyses suggested that hsa-miR-32-5p and hsa-miR-1246 levels were altered in critical versus asymptomatic individuals [28] and hsa-miR-200 could also affect *ACE2*/*TMPRSS2* expression (29). Here we have reported by functional analysis that hsa-miR-98-5p, hsa-miR-202-3p, hsa-miR-4458 and hsa-miR-4500 as the main relevant ones among others from hsa-let-7 family. Just one of them, hsa-miR-98-5p, has also been reported in targeting S*AR*S-CoV2 gene (ORF1ab) [30].

We described that several clinical parameters such as ferritin, D-dimer, LDH or CRP are good markers between mild and severe patients. Previous reports have also indicated high values of these biomarkers in combination with Absolute Neutrophil Count, Neutrophil to Lymphocyte Ratio (NLR) and Platelet to Lymphocyte ratio (PLR) as biomarkers associated with disease severity [31]. Moreover, we also found that age is a significant variable, age over 55 is associated with an increased risk of COVID-19 severe cases. Moreover, data published by H.Ashktorab et al.[32] confirmed our results, indicating that when analyses were adjusted for disease severity, significant variables were age over 65 years old, male sex, as well as having shortness of breath, elevated CRP and D-dimer. Although there are controversial data about the protection of influenza vaccine to COVID-19, here we did not find any representative protection role. Similarly, it was reported in studies of co-administration of influenza and COVID-19 vaccines with no reports in humoral response [33].

## Conclusion

To sum up, the inclusion of these three markers *ACE2* (rs2285666), *TMPRSS2* (rs2070788) and *MX1* genes (rs469390) in COVID-19 opens new strategies in the classification of these patients. CAA allele combination analysis of these SNPs was significantly associated with an increased risk of developing severe COVID-19. Similarly, G allele in *TMPRSS2* (rs2070788) in female cases was associated with an increased risk of developing severe COVID-19. We do a lot of emphasis on the use of *MX1, AR* and *ERG* biomarkers in gene expression analysis, due to most representative differences have been proved in these data. Moreover, a better understanding of molecular mechanisms of SARS-CoV-2 infection, could be used for an effective managing infection and inclusion of diagnostic and therapeutic biomarkers in clinical practice. However, we would like to include that one of the limitations of present results should be included and are related to limited sample size, but very well clinical and genetically classified. A deeper study increasing sample size will improve present data.

## Materials and methods

### Patients

Peripheral blood tubes collected in EDTA and Tempus™ RNA tubes were obtained from each patient. Samples were processed in the subsequent 4–6 h after collection, following a protocol depending on the subsequent analysis. Samples were frozen at -80ºC until future analysis.

All collected samples were confirmed in diagnosis of COVID-19 by RT-PCR and positive IgG serology. All of them follow-up inclusion criteria based on WHO classification. Inclusion criteria were revised periodically to update database trying to have balanced samples according to age, gender and severity. Those in mild disease were characterized by fever, malaise, cough, upper respiratory symptoms, and/or less common features of COVID-19 (headache, loss of taste or smell etc.). Moreover, patients in severe disease group fulfill the following features: (i) hypoxia: SPO_2_ ≤ 93% on atmospheric air or PaO_2_:FiO_2_ < 300 mmHg (SF ratio < 315); tachypnea: in respiratory distress or RR (respiratory rate) > 30 breaths/minutes; or more than 50% involvement seen on chest imaging [34].

A total of 329 samples (n = 186 mild and n = 143 severe) with a mean age of 55 (aged 33–80) years old were recruited from 2020 to 2022, all clinical data (ferritine, D dimer, CRP, troponin, and LDH); symptoms (fever, anosmia, asthenia, dyspnoea, long COVID, etc.); and intensive care unit (ICU) clinical follow-up (need of assisted ventilation, pneumonia, etc.), were included in the report, details described in Table 1.

The study protocol was approved by the Ethics Committee (CEI) with internal code 1329-N-21. Informed written consent from all participants was obtained in accordance with the tenets of the Declaration of Helsinki.

### DNA extraction

Genomic DNA extraction was obtained from whole blood of all samples, by using kit RealPure “SSS” (Durviz, Spain). DNA Quantification was performed by fluorescence using Qubit™3.0 (Invitrogen™ by Thermo Scientific, USA) and nanodrop 2000 system (Thermo Scientific, USA), this equipment was also used to check the 260/280 ratio as quality control. Extracted DNA was stored at − 20 °C until genotyping.

### RNA extraction

Total RNA from 258 plasma samples (n = 160 mild and n = 98 severe) of Tempus™ Blood RNA tubes were extracted using Tempus™ Spin RNA Isolation Kit protocol. Quality was validated by A260/A280 in NanoDrop™ 2000c. This analysis was performed in a sub-selection of samples to prove the role of present genes in expression patterns, just those samples with all clinical data were included for the analysis.

### Genotyping

SNPs selection was performed according to NCBI website in the most relevant data according COVID-19 and *TMPRSS2* published papers until 2022. Moreover, only those SNPs with an allelic frequency over 10% in minor allele (MAF) in Caucasian population were selected according to Ensembl database. We finally selected *ACE2* (rs2285666), *MX1* (rs469390) and *TMPRSS2* (rs2070788) for present analysis, see details of probes in Additional file 1: Tables S7 and S8.

DNA genotyping was performed using TaqMan® Genotyping Master Mix (Applied Biosystems, USA). Allelic discrimination assays were carried out in a 7900HT Fast Real-Time PCR System (Applied Biosystems, USA).

### Reverse transcription PCR and quantitative real‑time PCR (qPCR)

RNA reverse transcription was implemented using PrimeScript RT Reagent Kit (Takara Bio, JP). Quantitative polymerase chain reaction (qPCR) was performed with SYBR Green designed probes (Life Technologies, CA), for genes: *ERG, ETV5, AR, MX1;* and TaqMan™ gene expression assays (Thermofisher, USA) for: *ACE2* (Assay ID: Hs01085333_m1) and *TMPRSS2* (Assay ID: Hs01122322_m1) on a 96-wells plate with QuantStudio 6 Flex Real-Time PCR System (Applied Biosystems, USA). These genes were selected for having a relevant interaction with main genes of present article (*MX1, ACE2* and *TMPRSS2*). Primers were designed using Primer-Blast (NIH) software under the following conditions: span an exon-exon junction, PCR product size between 60–150 nucleotides and primer melting temperature within the range of 59–61 °C. Details of probes are in Additional file 1: Table S9. qPCR reactions were performed as follows: 95 °C during 10 min for enzyme activation; followed by 45 cycles of 15 s at 95 °C and 1 min at 60 °C for denaturing and annealing/extension.ineas All samples were run in triplicates, with a NTC (non-template control) in each plate. Threshold cycles (Ct) ≥ 35 were considered undetermined values. mRNAs expression levels were quantified using the comparative threshold cycle method (2 − ΔΔCt) relative to *HPRT1* (hypoxanthine phosphoribosyltransferase 1) expression as an endogenous control. Relative quantification parameter (RQ or 2 − ΔΔCt) was estimated for each case and used in statistical analysis. In order to differentiate between high and low expression levels, we took into account the median value of RQ for each gene. Values below the median value are in low expression; and values above the median value are in high expression for *ERG, ETV5, AR, MX1, ACE2* and *TMPRSS2*, see details in Additional file 1: Table S5.

### In silico* analysis*

An analysis of the different variants of present study was carried out in "The variant effect predictor" (https://www.ensembl.org/info/docs/tools/vep/index.html) [35]. This software was used to calculate changes in transcripts and malignancy of variants. We also used ClinVar tool (https://www.ncbi.nlm.nih.gov/clinvar/) for data validation.

A functional analysis of *TMPRSS2* and associated genes (*ACE2*, *ERG*, *AR*, *ETV5* and *MX1*) was performed. This analysis was achieved using IPA (Ingenuity Pathway Analysis) [29] and DAVID Bioinformatics Resources v6.8 (https://david.ncifcrf.gov/) to obtain the role of gene pool, clinical implication, ontology and involved metabolic pathways. Moreover, STRING search tool was used to represent a protein–protein interaction (PPI) network including our target genes (https://string-db.org/) [36]. GTEx was used to analyse the differential gene expression according to the presence of studied SNPs in *ACE2*, *MX1* and *TMPRSS2* in lung tissue. The data used for the analyses described in this manuscript were obtained from the GTEx Portal (https://gtexportal.org/home/faq#citePortal).

Finally, miRDB database (http://mirdb.org/) was used to predict the miRNAs and their targeted genes, and the network image was obtained using the miRNet online tool (https://www.mirnet.ca) [37]. This analysis was performed using the default parameters, and *Homo sapiens* was selected as the specific taxonomy.

### Statistical analysis

Hardy–Weinberg equilibrium (HWE) was performed using the online SNPstats and Metagenyo tools [38]. SPSS v.22 software package (IBM Corporation, USA) was used for statistical analyses. The association between COVID-19 severity, as well as clinical outputs and SNPs were analysed by chi-square test (χ2) or Fisher exact test for a small sample size. Binary logistic regression analyses using different genetic models (codominant, dominant and recessive), were used to assess which of the genetic factor might be determinant in aggressiveness.

To analyse clinical variables (ferritin, D-dimer, CRP, troponin, lactate dehydrogenase and IL-6), we divided each one into two groups of data in order to their values (low or high) and compared with single SNPs. To determine better contribution of the SNPs to COVID-19 aggressiveness, associations between SNPs were generated using the online SNPstats software.

Gene expression analysis was performed using non-parametric test (U-Mann Whitney) for all variables. Shapiro-Wilks test revealed that our results did not follow a gauss distribution, so we used U- Mann Whitney. Statistical significance level was *p* < 0.05.

## Supplementary Information

**Additional file 1.** Supplementray tables and figures.

## Data Availability

The materials that support the conclusion of this article have been included within the article.

## Abbreviations

*ACE2*: Angiotensin converting enzyme 2
ARNTL: Aryl hydrocarbon receptor nuclear translocator like
*BST2*: Bone marrow stromal cell antigen 2
CD-147: Cluster of differentiation 147
CI: Confidence interval
COVID: Coronavirus disease
CRP: C-reactive protein
*DPP4*: Dipeptidyl peptidase 4
EA: Effect allele
EAF: Effect allele frequency
EDTA: Ethylene diamine tetra acetate
HGF: Hepatocyte growth factor
HWE: Hardy–Weinberg equilibrium
ICU: Intensive care unit
*IFI6*: Interferon alpha inducible protein 6
*IFI27*: Interferon alpha inducible protein 27
*IFI35*: Interferon induced protein 35
*IFIT1*: Interferon Induced protein with tetratricopeptide repeats 1
*IFITM3*: Interferon induced transmembrane protein 3
IFN: Interferon
IL-6: Interleukin 6
IPA: Ingenuity pathway analysis
LDH: Lactate dehydrogenase
MAF: Minor allele frequency
MDSC: Myeloid-derived suppressor cell
*MX1*: MX dynamin like GTPase 1
*MX2*: MX dynamin like GTPase 2
NLR: Neutrophil to lymphocyte ratio
NMD: Nonsense mediated decay
*OAS1*: 2′-5′-Oligoadenylate synthetase 1
*OAS2*: 2′-5′-Oligoadenylate synthetase 2
OAT: Ornithine aminotransferase
OR: Odds ratio
P2X7: Purinergic receptor P2X 7
PD-1: Programmed death 1 protein
PD-L1: Programmed death-ligand 1
PLR: Platelet to lymphocyte ratio
PPI: Protein–protein interaction
RAS: Renin-angiotensin system
*RSAD2*: Radical S-adenosyl methionine domain containing 2
SARS-CoV-2: Severe acute respiratory syndrome-coronavirus-2
SNP: Single nucleotide polymorphism
SP9: Sp9 transcription factor
*STAT2*: Signal transducer and activator of transcription 2
*TMPRSS2*: Transmembrane serine protease 2
VEP: Variant effect predictor
WARS: Tryptophan-tRNA ligase
