# Supplementary material for: Analyzing the role of ACE2, AR, MX1 and TMPRSS2 genetic markers for COVID-19 severity
Source: Hum Genomics. 2023 Jun 7;17:50. doi: 10.1186/s40246-023-00496-2 (PMC10245351; doi:10.1186/s40246-023-00496-2)
Supplement: Supplementary file 1 — Additional file 1. Supplementray tables and figures. [file 40246_2023_496_MOESM1_ESM.doc]

**Supplementary Information**

**Table S1.** Values of clinical data.

|  | **Increased ferritin (ng/mL)** | **D-dimer (ng/mL)** | **CRP (mg/L)** | **Troponin (ng/L)** | **LDH (U/L)** | **IL-6 (pg/mL)** |
| --- | --- | --- | --- | --- | --- | --- |
| **Sample (N)** | 215 | 193 | 214 | 49 | 189 | 19 |
| **Missing data** | 129 | 151 | 130 | 295 | 155 | 325 |
| **Median (IQR)** | 258 (156.55) | 4.77 (4.09) | 19.8 (18) | 9 (4) | 262 (85) | 51 (35.5) |

**Table S2**. Distribution of genotypes in clinical data for asymptomatic/slight disease and severe/critical disease.

|  |  | | | | | | | | | | |  | | | | |  | | | | | |  | | | | |  | | | | |
| --- | --- | --- | --- | --- | --- | --- | --- | --- | --- | --- | --- | --- | --- | --- | --- | --- | --- | --- | --- | --- | --- | --- | --- | --- | --- | --- | --- | --- | --- | --- | --- | --- |
|  | **Asymptomatic/mild disease**  **n (%)** | | | | | | | | | | |  | | | | | **Severe/critical disease**  **n (%)** | | | | | | | | | | |  | | | | |
| **Ferritin** | **Low**  **n (%)** | | | | | | | **High**  **n (%)** | | | | ***p*-value*** | | | | | **Low**  **n (%)** | | | | | | **High**  **n (%)** | | | | | ***p*-value*** | | | | |
| ***MX1*** | | | | | | | | | | | | 0.092 | | | | |  | | | | | |  | | | | | 0.985 | | | | |
| AA | 19 (26.4%) | | | | | | | 14 (33.3%) | | | |  | | | | | 10 (32.3%) | | | | | | 20 (30.8%) | | | | |  | | | | |
| AG | 44 (61.1%) | | | | | | | 17 (40.5%) | | | |  | | | | | 15 (48.4%) | | | | | | 34 (52.3%) | | | | |  | | | | |
| GG | 9 (12.5%) | | | | | | | 11 (26.2%) | | | |  | | | | | 6 (19.4%) | | | | | | 11 (16.9%) | | | | |  | | | | |
| ***ACE2*** | | | | | | | | | | | | 0.177 | | | | |  | | | | | |  | | | | | 0.430 | | | | |
| CC | 50 (69.4%) | | | | | | | 36 (85.7%) | | | |  | | | | | 19 (61.3%) | | | | | | 50 (76.9%) | | | | |  | | | | |
| CT | 16 (22.2%) | | | | | | | 4 (9.5%) | | | |  | | | | | 7 (22.6%) | | | | | | 10 (15.4%) | | | | |  | | | | |
| TT | 6 (8.3%) | | | | | | | 2 (4.8%) | | | |  | | | | | 5 (16.1%) | | | | | | 5 (7.7%) | | | | |  | | | | |
| ***TMPRSS2*** | | | | | | | | | | | | 0.554 | | | | |  | | | | | |  | | | | | 0.992 | | | | |
| AA | 26 (36.1%) | | | | | | | 12 (28.6%) | | | |  | | | | | 10 (32.3%) | | | | | | 21 (32.3%) | | | | |  | | | | |
| AG | 31 (43.1%) | | | | | | | 22 (52.4%) | | | |  | | | | | 15 (48.4%) | | | | | | 33 (50.8%) | | | | |  | | | | |
| GG | 15 (20.8%) | | | | | | | 8 (19%) | | | |  | | | | | 6 (19.4%) | | | | | | 11 (16.9%) | | | | |  | | | | |
|  | | **Asymptomatic/mild disease**  **n (%)** | | | | | | | | | | |  | | | | | **Severe/critical disease**  **n (%)** | | | | | | | | | |  | | | | |
| **D-Dimer** | | **Low**  **n (%)** | | | | | | | **High**  **n (%)** | | | | ***p*-value*** | | | | | **Low**  **n (%)** | | | | | **High**  **n (%)** | | | | | ***p*-value*** | | | | |
| ***MX1*** | | | | | | | | | | | | | 0.890 | | | | |  | | | | |  | | | | | 0.417 | | | | |
| AA | | 13 (31.7%) | | | | | | | 20 (31.7%) | | | |  | | | | | 15 (29.4%) | | | | | 11 (29.7%) | | | | |  | | | | |
| AG | | 23 (56.1%) | | | | | | | 32 (50.8%) | | | |  | | | | | 29 (56.9%) | | | | | 16 (43.3%) | | | | |  | | | | |
| GG | | 5 (12.2%) | | | | | | | 11 (17.5%) | | | |  | | | | | 7 (13.7%) | | | | | 10 (27%) | | | | |  | | | | |
| ***ACE2*** | | | | | | | | | | | | | 0.952 | | | | |  | | | | |  | | | | | 0.622 | | | | |
| CC | | 31 (75.6%) | | | | | | | 49 (77.7%) | | | |  | | | | | 40 (78.4%) | | | | | 25 (67.6%) | | | | |  | | | | |
| CT | | 8 (19.5%) | | | | | | | 10 (15.9%) | | | |  | | | | | 6 (11.8%) | | | | | 8 (21.6%) | | | | |  | | | | |
| TT | | 2 (4.9%) | | | | | | | 4 (6.3%) | | | |  | | | | | 5 (9.8%) | | | | | 4 (10.8%) | | | | |  | | | | |
| ***TMPRSS2*** | | | | | | | | | | | | | 0.601 | | | | |  | | | | |  | | | | | 0.368 | | | | |
| AA | | 16 (39%) | | | | | | | 17 (27%) | | | |  | | | | | 13 (25.5%) | | | | | 15 (40.5%) | | | | |  | | | | |
| AG | | 18 (43.9%) | | | | | | | 31 (49.2%) | | | |  | | | | | 30 (58.8%) | | | | | 15 (40.5%) | | | | |  | | | | |
| GG | | 7 (17.1%) | | | | | | | 15 (23.8%) | | | |  | | | | | 8 (15.7%) | | | | | 7 (19%) | | | | |  | | | | |
|  | | | | | **Asymptomatic/mild disease**  **n (%)** | | | | | | | | |  | | | | | **Severe/critical disease**  **n (%)** | | | | | | | | |  | | | | |
| **PCR** | | | | | **Low**  **n (%)** | | | | | **High**  **n (%)** | | | | ***p*-value*** | | | | | **Low**  **n (%)** | | | | | **High**  **n (%)** | | | | ***p*-value*** | | | | |
| ***MX1*** | | | | | | | | | | | | | | 0.741 | | | | |  | | | | |  | | | | 0.238 | | | | |
| AA | | | | | 22 (30.1%) | | | | | 12 (31.6%) | | | |  | | | | | 9 (29%) | | | | | 21 (32.3%) | | | |  | | | | |
| AG | | | | | 38 (52.1%) | | | | | 22 (57.9%) | | | |  | | | | | 13 (41.9%) | | | | | 35 (53.8%) | | | |  | | | | |
| GG | | | | | 13 (17.8%) | | | | | 4 (10.5%) | | | |  | | | | | 9 (29%) | | | | | 9 (13.8%) | | | |  | | | | |
| ***ACE2*** | | | | | | | | | | | | | | 0.148 | | | | |  | | | | |  | | | | 0.378 | | | | |
| CC | | | | | 59 (80.8%) | | | | | 26 (68.4%) | | | |  | | | | | 20 (64.5%) | | | | | 51 (78.5%) | | | |  | | | | |
| CT | | | | | 9 (12.3%) | | | | | 11 (28.9%) | | | |  | | | | | 7 (22.6%) | | | | | 9 (13.8%) | | | |  | | | | |
| TT | | | | | 5 (6.8%) | | | | | 1 (2.6%) | | | |  | | | | | 4 (12.9%) | | | | | 5 (7.7%) | | | |  | | | | |
| ***TMPRSS2*** | | | | | | | | | | | | | | 0.796 | | | | |  | | | | |  | | | | 0.327 | | | | |
| AA | | | | | 24 (32.9%) | | | | | 11 (28.9%) | | | |  | | | | | 13 (41.9%) | | | | | 17 (26.1%) | | | |  | | | | |
| AG | | | | | 32 (43.8%) | | | | | 20 (52.6%) | | | |  | | | | | 13 (41.9%) | | | | | 36 (55.4%) | | | |  | | | | |
| GG | | | | | 17 (23.3%) | | | | | 7 (18.4%) | | | |  | | | | | 5 (16.2%) | | | | | 12 (18.5%) | | | |  | | | | |
|  | | **Asymptomatic/mild disease**  **n (%)** | | | | | | | | | |  | | | | | **Severe/critical disease**  **n (%)** | | | | | | | | | | |  | | | | |
| **Troponin** | | **Low**  **n (%)** | | | | | | **High**  **n (%)** | | | | ***p*-value*** | | | | | **Low**  **n (%)** | | | | | **High**  **n (%)** | | | | | | ***p*-value*** | | | | |
| ***MX1*** | | | | | | | | | | | | **0.042** | | | | |  | | | | |  | | | | | | 0.907 | | | | |
| AA | | 2 (18.2%) | | | | | | 8 (57.1%) | | | |  | | | | | 4 (26.7%) | | | | | 8 (25.8%) | | | | | |  | | | | |
| AG | | 6 (54.5%) | | | | | | 6 (42.9%) | | | |  | | | | | 8 (53.3%) | | | | | 15 (48.8%) | | | | | |  | | | | |
| GG | | 3 (27.3%) | | | | | | 0 (0%) | | | |  | | | | | 3 (20%) | | | | | 8 (25.8%) | | | | | |  | | | | |
| ***ACE2*** | | | | | | | | | | | | 0.319 | | | | |  | | | | |  | | | | | | 0.309 | | | | |
| CC | | 6 (54.5%) | | | | | | 11 (78.6%) | | | |  | | | | | 14 (93.3%) | | | | | 24 (77.4%) | | | | | |  | | | | |
| CT | | 4 (36.4%) | | | | | | 3 (21.4%) | | | |  | | | | | 0 (0%) | | | | | 4 (12.9%) | | | | | |  | | | | |
| TT | | 1 (9.1%) | | | | | | 0 (0%) | | | |  | | | | | 1 (6.7%) | | | | | 3 (9.7%) | | | | | |  | | | | |
| ***TMPRSS2*** | | | | | | | | | | | | **0.040** | | | | |  | | | | |  | | | | | | 0.455 | | | | |
| AA | | 3 (27.3%) | | | | | | 3 (21.4%) | | | |  | | | | | 4 (26.7%) | | | | | 14 (45.2%) | | | | | |  | | | | |
| AG | | 8(72.7%) | | | | | | 5 (35.7%9 | | | |  | | | | | 9 (60%) | | | | | 13 (41.9%) | | | | | |  | | | | |
| GG | | 0 (0%) | | | | | | 6 (42.9%) | | | |  | | | | | 2 (13.3%) | | | | | 4 (12.9%) | | | | | |  | | | | |
|  | **Asymptomatic/mild disease**  **n (%)** | | | | | | | | | | | |  | | | | | **Severe/critical disease**  **n (%)** | | | | | | | | | | | |  | | |
| **LDH** | **Low**  **n (%)** | | | | | | | | **High**  **n (%)** | | | | ***p*-value*** | | | | | **Low**  **n (%)** | | | | | | | **High**  **n (%)** | | | | | ***p*-value*** | | |
| ***MX1*** | | | | | | | | | | | | | 0.981 | | | | |  | | | | | | |  | | | | | 0.667 | | |
| AA | 18 (30%) | | | | | | | | 10 (30.3%) | | | |  | | | | | 10 (30.3%) | | | | | | | 20 (36.4%) | | | | |  | | |
| AG | 33 (55%) | | | | | | | | 18 (54.5%) | | | |  | | | | | 17 (51.5%) | | | | | | | 25 (45.5%) | | | | |  | | |
| GG | 9 (15%) | | | | | | | | 5 (15.1%) | | | |  | | | | | 6 (18.2%) | | | | | | | 10 (18.2%) | | | | |  | | |
| ***ACE2*** | | | | | | | | | | | | | 0.190 | | | | |  | | | | | | |  | | | | | 0.071 | | |
| CC | 47 (78.3%) | | | | | | | | 23 (69.7%) | | | |  | | | | | 19 (57.6%) | | | | | | | 44 (80%) | | | | |  | | |
| CT | 7 (11.7%) | | | | | | | | 9 (27.3%) | | | |  | | | | | 8 (24.2%) | | | | | | | 8 (14.5%) | | | | |  | | |
| TT | 6 (10%) | | | | | | | | 1 (3%) | | | |  | | | | | 6 (18.2%) | | | | | | | 3 (5.5%) | | | | |  | | |
| ***TMPRSS2*** | | | | | | | | | | | | | 0.713 | | | | |  | | | | | | |  | | | | | 0.472 | | |
| AA | 21 (35%) | | | | | | | | 9 (27.3%) | | | |  | | | | | 11 (33.3%) | | | | | | | 17 (30.9%) | | | | |  | | |
| AG | 28 (46.7%) | | | | | | | | 15 (45.5%) | | | |  | | | | | 18 (54.5%) | | | | | | | 26 (47.3%) | | | | |  | | |
| GG | 11 (18.3%) | | | | | | | | 9 (27.3%) | | | |  | | | | | 4 (12.1%) | | | | | | | 12 (21.8%) | | | | |  | | |
|  | | **Asymptomatic/mild disease**  **n (%)** | | | | | | | | | |  | | | | | **Severe/critical disease**  **n (%)** | | | | | | | | | |  | | | | | |
| **IL6** | | **Low**  **n(%)** | | | | | | **High**  **n (%)** | | | | ***p*-value*** | | | | | **Low**  **n (%)** | | | | | | **High**  **n (%)** | | | | ***p*-value*** | | | | | |
| ***MX1*** | | | | | | | | | | | | 0.405 | | | | |  | | | | | |  | | | | 0.255 | | | | | |
| AA | | 2 (50%) | | | | | | 1 (16.7%) | | | |  | | | | | 4 (66.7%) | | | | | | 1 (20%) | | | |  | | | | | |
| AG | | 1 (25%) | | | | | | 4 (66.7%) | | | |  | | | | | 1 (16.7%) | | | | | | 3 (60%9 | | | |  | | | | | |
| GG | | 1 (25%9 | | | | | | 1 (16.7%) | | | |  | | | | | 1 (16.7%) | | | | | | 1 (20%) | | | |  | | | | | |
| ***ACE2*** | | | | | | | | | | | | 0.133 | | | | |  | | | | | |  | | | | - | | | | | |
| CC | | 2 (50%) | | | | | | 6 (100%) | | | |  | | | | | 5 (83.3%) | | | | | | 4 (80%) | | | |  | | | | | |
| CT | | 2 (50%) | | | | | |  | | | |  | | | | | 1 (16.7%) | | | | | | 0 (0%) | | | |  | | | | | |
| TT | |  | | | | | |  | | | |  | | | | | 0 (0%) | | | | | | 1 (20%) | | | |  | | | | | |
| ***TMPRSS2*** | | | | | | | | | | | | 0.153 | | | | |  | | | | | |  | | | | 0.535 | | | | | |
| AA | | 2 (50%) | | | | | | 1 (16.7%) | | | |  | | | | | 3 (50%) | | | | | | 1 (20%) | | | |  | | | | | |
| AG | | 1 (25%) | | | | | | 5 (83.3%) | | | |  | | | | | 1 (16.7%) | | | | | | 2 (40%) | | | |  | | | | | |
| GG | | 1 (25%) | | | | | | 0 (0%) | | | |  | | | | | 2 (33.3%) | | | | | | 2 (40%) | | | |  | | | | | |
|  | | | | **Asymptomatic/mild disease**  **n (%)** | | | | | | | | | | | |  | | | | | **Severe/critical disease**  **n (%)** | | | | | | | |  | | |  |
| **Artificial respiration** | | | | **No**  **n (%)** | | | | | **Yes**  **n (%)** | | | | | | | ***p*-value*** | | | | | **No**  **n (%)** | | | | **Yes**  **n (%)** | | | | ***p*-value*** | | |  |
| ***MX1*** | | | | | | | | | | | | | | | | 0.519 | | | | |  | | | |  | | | | 0.623 | | |  |
| AA | | | | 3 (37.5%) | | | | | 6 (37.5%) | | | | | | |  | | | | | 13 (31.7%) | | | | 18 (30%) | | | |  | | |  |
| AG | | | | 2 (25%) | | | | | 7 (43.7%) | | | | | | |  | | | | | 23 (56.1%) | | | | 33 (55%) | | | |  | | |  |
| GG | | | | 3 (37.5%) | | | | | 3 (18.8%) | | | | | | |  | | | | | 5 (12.2%) | | | | 9 (15%) | | | |  | | |  |
| ***ACE2*** | | | | | | | | | | | | | | | | 0.475 | | | | |  | | | |  | | | | 0.774 | | |  |
| CC | | | | 7 (87.5%) | | | | | 12 (75%) | | | | | | |  | | | | | 15 (78.9%) | | | | 33 (80.5%) | | | |  | | |  |
| CT | | | | 1 (12.5%) | | | | | 4 (25%) | | | | | | |  | | | | | 2 (10.5%) | | | | 5 (12.2%) | | | |  | | |  |
| TT | | | | - | | | | | - | | | | | | |  | | | | | 2 (10.5%) | | | | 3 (7.3%) | | | |  | | |  |
| ***TMPRSS2*** | | | | | | | | | | | | | | | | 0.781 | | | | |  | | | |  | | | | **0.027** | | |  |
| AA | | | | 2 (25%) | | | | | 5 (31.3%) | | | | | | |  | | | | | 8 (42.1%) | | | | 11 (26.8%) | | | |  | | |  |
| AG | | | | 4 (50%) | | | | | 7 (43.7%) | | | | | | |  | | | | | 5 (26.3%) | | | | 26 (63.4%) | | | |  | | |  |
| GG | | | | 2 (25%) | | | | | 4 (25%) | | | | | | |  | | | | | 6 (31.6%) | | | | 4 (9.8%) | | | |  | | |  |
|  | | | | | | | **Asymptomatic/mild disease**  **n (%)** | | | | | | | |  | | | | | **Severe/critical disease**  **n (%)** | | | | | |  | | | | |  | |
| **Systemic inflammatory response** | | | | | | | **No**  **n (%)** | | **Yes**  **n (%)** | | | | | | ***p*-value*** | | | | | **No**  **n (%)** | | | **Yes**  **n (%)** | | | ***p*-value*** | | | | |  | |
| ***MX1*** | | | | | | | | | | | | | | | 0.426 | | | | |  | | |  | | | 0.183 | | | | |  | |
| AA | | | | | | | 1 (25%) | | 5 (62.5%) | | | | | |  | | | | | 0 (0%) | | | 5 (25%) | | |  | | | | |  | |
| AG | | | | | | | 2 (50%) | | 2 (25%) | | | | | |  | | | | | 8 (72.7%) | | | 11 (55%) | | |  | | | | |  | |
| GG | | | | | | | 1 (25%) | | 1 (12.5%) | | | | | |  | | | | | 3 (27.3%) | | | 3 (15%) | | |  | | | | |  | |
| ***ACE2*** | | | | | | | | | | | | | | | 0.926 | | | | |  | | |  | | | 0.551 | | | | |  | |
| CC | | | | | | | 3 (75%) | | 6 (75%) | | | | | |  | | | | | 9 (81.8%) | | | 12 (63.2%) | | |  | | | | |  | |
| CT | | | | | | | 1 (25%) | | 2 (25%) | | | | | |  | | | | | 0 (0%) | | | 5 (26.3%) | | |  | | | | |  | |
| TT | | | | | | | 0 (0%) | | 0 (0%) | | | | | |  | | | | | 2 (18.2%) | | | 2 (10.5%) | | |  | | | | |  | |
| ***TMPRSS2*** | | | | | | | | | | | | | | | 0.852 | | | | |  | | |  | | | 0.802 | | | | |  | |
| AA | | | | | | | 1 (25%) | | 1 (12.5%) | | | | | |  | | | | | 4 (36.4%) | | | 6 (31.5%) | | |  | | | | |  | |
| AG | | | | | | | 2 (50%) | | 4 (50%) | | | | | |  | | | | | 6 (54.5%) | | | 11 (55%) | | |  | | | | |  | |
| GG | | | | | | | 1 (25%) | | 3 (37.5%) | | | | | |  | | | | | 1 (9.1%) | | | 2 (10.5%) | | |  | | | | |  | |
|  | | | **Asymptomatic/mild disease**  **n (%)** | | | | | | | | | |  | | | | | **Severe/critical disease**  **n (%)** | | | | | | | | |  | | | | | |
| **Influenza vaccine** | | | **No**  **n (%)** | | | | | | **Yes**  **n (%)** | | | | ***p*-value*** | | | | | **No**  **n (%)** | | | | | **Yes**  **n (%)** | | | | ***p*-value*** | | | | | |
| ***MX1*** | | | | | | | | | | | | | **0.038** | | | | |  | | | | |  | | | | 0.375 | | | | | |
| AA | | | 23 (25.6%) | | | | | | 10 (27.7%) | | | |  | | | | | 10 (31.3%) | | | | | 10 (27%) | | | |  | | | | | |
| AG | | | 52 (57.8%) | | | | | | 15 (41.7%) | | | |  | | | | | 18 (56.3%) | | | | | 17 (46%) | | | |  | | | | | |
| GG | | | 15 (16.7%) | | | | | | 11 (30.5%) | | | |  | | | | | 4 (12.5%) | | | | | 10 (27%) | | | |  | | | | | |
| ***ACE2*** | | | | | | | | | | | | | **0.019** | | | | |  | | | | |  | | | | 0.637 | | | | | |
| CC | | | 70 (77.8%) | | | | | | 23 (63.9%) | | | |  | | | | | 19 (59.4%) | | | | | 24 (64.9%) | | | |  | | | | | |
| CT | | | 12 (13.3%) | | | | | | 11 (30.5%) | | | |  | | | | | 8 (25%) | | | | | 6 (16.2%9 | | | |  | | | | | |
| TT | | | 8 (8.9%) | | | | | | 2 (5.6%) | | | |  | | | | | 5 (15.6%) | | | | | 7 (18.9%) | | | |  | | | | | |
| ***TMPRSS2*** | | | | | | | | | | | | | 0.058 | | | | |  | | | | |  | | | | 0.330 | | | | | |
| AA | | | 34 (37.8%) | | | | | | 14 (38.9%) | | | |  | | | | | 10 (31.3%) | | | | | 12 (32.4%) | | | |  | | | | | |
| AG | | | 38 (42.2%) | | | | | | 19 (52.8%) | | | |  | | | | | 14 (43.8%) | | | | | 21 (56.8%) | | | |  | | | | | |
| GG | | | 18 (20%) | | | | | | 3 (8.3%) | | | |  | | | | | 8 (25%) | | | | | 4 (10.8%) | | | |  | | | | | |
|  | | | | | | **Asymptomatic/mild disease**  **n (%)** | | | | | | |  | | | | | **Severe/critical disease**  **n (%)** | | | | | | | | |  | | | | | |
| **Days hospitalized** | | | | | | **< 37**  **n (%)** | | | | | **≥37**  **n (%)** | | ***p*-value*** | | | | | **<37**  **n (%)** | | | | | **≥37**  **n (%)** | | | | ***p*-value*** | | | | | |
| ***MX1*** | | | | | | | | | | | | | 0.161 | | | | |  | | | | |  | | | | 0.251 | | | | | |
| AA | | | | | | 7 (36.8%) | | | | | 2 (50%) | |  | | | | | 12 (26.1%) | | | | | 2 (25%) | | | |  | | | | | |
| AG | | | | | | 8 (42.1%) | | | | | 1 (25%) | |  | | | | | 26 (56.5%) | | | | | 5 (52.5%) | | | |  | | | | | |
| GG | | | | | | 4 (21%) | | | | | 1 (25%) | |  | | | | | 8 (17.4%) | | | | | 1 (12.5%) | | | |  | | | | | |
| ***ACE2*** | | | | | | | | | | | | | 0.179 | | | | |  | | | | |  | | | | 0.402 | | | | | |
| CC | | | | | | 15 (78.9%) | | | | | 3 (75%) | |  | | | | | 34 (73.9%) | | | | | 7 (87.5%) | | | |  | | | | | |
| CT | | | | | | 4 (21.1%) | | | | | 1 (25%) | |  | | | | | 8 (17.4%) | | | | | 0 (0%) | | | |  | | | | | |
| TT | | | | | | 0 (0%) | | | | | 0 (0%) | |  | | | | | 4 (8.7%) | | | | | 1 (12.5%) | | | |  | | | | | |
| ***TMPRSS2*** | | | | | | | | | | | | | 0.305 | | | | |  | | | | |  | | | | 0.367 | | | | | |
| AA | | | | | | 5 (26.3%) | | | | | 1 (25%) | |  | | | | | 13 (28.3%) | | | | | 4 (50%) | | | |  | | | | | |
| AG | | | | | | 8 (42.1%) | | | | | 2 (50%) | |  | | | | | 24 (52.2%) | | | | | 4 (50%) | | | |  | | | | | |
| GG | | | | | | 6 (31.6%) | | | | | 1 (25%) | |  | | | | | 9 (19.6%) | | | | | 0 (0%) | | | |  | | | | | |
|  | | | | | | **Asymptomatic/mild disease**  **n (%)** | | | | | | |  | | | | | **Severe/critical disease**  **n (%)** | | | | | | | | |  | | | | | |
| **Long-COVID** | | | | | | **No**  **n (%)** | | | | | **Yes**  **n (%)** | | ***p*-value*** | | | | | **No**  **n (%)** | | | | | **Yes**  **n (%)** | | | | ***p*-value*** | | | | | |
| ***MX1*** | | | | | | | | | | | | | 0.980 | | | | |  | | | | |  | | | | 0.881 | | | | | |
| AA | | | | | | 9 (34.6%) | | | | | 6 (37.5%) | |  | | | | | 2 (14.3%) | | | | | 2 (14.3%) | | | |  | | | | | |
| AG | | | | | | 10 (38.5%) | | | | | 6 (37.5%) | |  | | | | | 10 (71.4%) | | | | | 9 (64.3%) | | | |  | | | | | |
| GG | | | | | | 7 (26.9%) | | | | | 4 (25%) | |  | | | | | 2 (14.3%) | | | | | 3 (21.4%) | | | |  | | | | | |
| ***ACE2*** | | | | | | | | | | | | | 0.239 | | | | |  | | | | |  | | | | 0.269 | | | | | |
| CC | | | | | | 21 (80.8%) | | | | | 13 (81.3%) | |  | | | | | 12 (85.7%) | | | | | 9 (64.3%) | | | |  | | | | | |
| CT | | | | | | 2 (7.7%) | | | | | 3 (18.8%) | |  | | | | | 2 (14.3%) | | | | | 3 (21.4%) | | | |  | | | | | |
| TT | | | | | | 3 (11.5%) | | | | | 0 (0%) | |  | | | | | 0 (0%) | | | | | 2 (14.3%) | | | |  | | | | | |
| ***TMPRSS2*** | | | | | | | | | | | | | 0.688 | | | | |  | | | | |  | | | | 0.670 | | | | | |
| AA | | | | | | 12 (46.2%) | | | | | 8 (50%) | |  | | | | | 4 (28.6%) | | | | | 6 (42.9%) | | | |  | | | | | |
| AG | | | | | | 8 (30.8%) | | | | | 6 (37.5%) | |  | | | | | 8 (57.1%) | | | | | 7 (50%) | | | |  | | | | | |
| GG | | | | | | 6 (23.1%) | | | | | 2 (12.5%) | |  | | | | | 2 (14.3%) | | | | | 1 (7.1%) | | | |  | | | | | |

*Pearson's chi-squared test (χ^2^)

In bold statistically significant values.

**Table S3.** Binary logistic regression model for clinical data in mild and severe patients.

| **Influenza vaccine** | **No** | | | |  | **Yes** | | | |
| --- | --- | --- | --- | --- | --- | --- | --- | --- | --- |
|  | **Asymptomatic/mild disease**  **n (%)** | **Severe/critical disease**  **n(%)** | **OR (95% CI)** | **p-value*** |  | **Asymptomatic/mild disease**  **n (%)** | **Severe/critical disease**  **n(%)** | **OR (95% CI)** | **p-value*** |
| ***MX1*** |  |  |  |  |  |  |  |  |  |
| AA | 23 (25.6%) | 10 (31.3%) | Ref. |  |  | 10 (27.7%) | 10 (27%) | Ref. |  |
| AG | 52 (57.8%) | 18 (56.3%) | 0.79 (0.32-1.99) | 0.630 |  | 15 (41.7%) | 17 (46%) | 1.13 (0.37 -3.47) | 0.826 |
| GG | 15 (16.7%) | 4 (12.5%) | 0.61 (0.16-2.31) | 0.470 |  | 11 (30.5%) | 10 (27%) | 0.91 (0.27-3.10) | 0.879 |
| ***ACE2*** |  |  |  |  |  |  |  |  |  |
| CC | 70 (77.8%) | 19 (59.4%) | Ref. |  |  | 23 (63.9%) | 24 (64.9%) | Ref. |  |
| CT | 12 (13.3%) | 8 (25%) | 2.45 (0.87-6.86) | 0.087 |  | 11 (30.5%) | 6 (16.2%9 | 0.53 (0.17-1.65) | 0.268 |
| TT | 8 (8.9%) | 5 (15.6%) | 2.30 (0.67-7.85) | 0.183 |  | 2 (5.6%) | 7 (18.9%) | 3.36 (0.63-17.86) | 0.156 |
| ***TMPRSS2*** |  |  |  |  |  |  |  |  |  |
| AA | 34 (37.8%) | 10 (31.3%) | Ref. |  |  | 14 (38.9%) | 12 (32.4%) | Ref. |  |
| AG | 38 (42.2%) | 14 (43.8%) | 1.25 (0.49-3.18) | 0.636 |  | 19 (52.8%) | 21 (56.8%) | 1.29 (0.48-3.47) | 0.517 |
| GG | 18 (20%) | 8 (25%) | 1.51 (0.51-4.50) | 0.458 |  | 3 (8.3%) | 4 (10.8%) | 1.56 (0.29-8.38) | 0.607 |

| **Artificial respiration** | **No** | | | |  | **Yes** | | | |
| --- | --- | --- | --- | --- | --- | --- | --- | --- | --- |
|  | **Asymptomatic/mild disease**  **n (%)** | **Severe/critical disease**  **n(%)** | **OR (95% CI)** | **p-value*** |  | **Asymptomatic/mild disease**  **n (%)** | **Severe/critical disease**  **n(%)** | **OR (95% CI)** | **p-value*** |
| ***MX1*** |  |  |  |  |  |  |  |  |  |
| AA | 3 (37.5%) | 13 (31.7%) | Ref. |  |  | 6 (37.5%) | 18 (30%) | Ref. |  |
| AG | 2 (25%) | 23 (56.1%) | 3.00 (0.37-24.17) | 0.302 |  | 7 (43.7%) | 33 (55%) | 1.51 (0.42-5.48) | 0.525 |
| GG | 3 (37.5%) | 5 (12.2%) | 0.80 (0.10-6.35) | 0.101 |  | 3 (18.8%) | 9 (15%) | 0.77 (0.14-4.33) | 0.766 |
| ***ACE2*** |  |  |  |  |  |  |  |  |  |
| CC | 7 (87.5%) | 15 (78.9%) | Ref. |  |  | 12 (75%) | 33 (80.5%) | Ref. |  |
| CT | 1 (12.5%) | 2 (10.5%) | 0.93 (0.07-12.10) | 0.958 |  | 4 (25%) | 5 (12.2%) | 0.46 (0.10-1.98) | 0.294 |
| TT | - | 2 (10.5%) | - | - |  | - | 3 (7.3%) | - | - |
| ***TMPRSS2*** |  |  |  |  |  |  |  |  |  |
| AA | 2 (25%) | 8 (42.1%) | Ref. |  |  | 5 (31.3%) | 11 (26.8%) | Ref. |  |
| AG | 4 (50%) | 5 (26.3%) | 0.31 (0.04-2.38) | 0.262 |  | 7 (43.7%) | 26 (63.4%) | 1.69 (0.44-6.50) | 0.446 |
| GG | 2 (25%) | 6 (31.6%) | 0.75 (0.08-6.96) | 0.800 |  | 4 (25%) | 4 (9.8%) | 0.46 (0.08-2.60) | 0.375 |
|  |  |  |  |  |  |  |  |  |  |
| **Troponin** | **Low** | | | |  | **High** | | | |
|  | **Asymptomatic/mild disease**  **n (%)** | **Severe/critical disease**  **n(%)** | **OR (95% CI)** | **p-value*** |  | **Asymptomatic/mild disease**  **n (%)** | **Severe/critical disease**  **n(%)** | **OR (95% CI)** | **p-value*** |
| ***MX1*** |  |  |  |  |  |  |  |  |  |
| AA | 2 (18.2%) | 4 (26.7%) | Ref. |  |  | 8 (57.1%) | 8 (25.8%) | Ref. |  |
| AG | 6 (54.5%) | 8 (53.3%) | 0.67 (0.09-4.93) | 0.691 |  | 6 (42.9%) | 15 (48.8%) | 2.50 (0.64-9.76) | 0.188 |
| GG | 3 (27.3%) | 3 (20%) | 0.50 (0.05-5.15) | 0.500 |  | 0 (0%) | 8 (25.8%) | - |  |
| ***ACE2*** |  |  |  |  |  |  |  |  |  |
| CC | 6 (54.5%) | 14 (93.3%) | Ref. |  |  | 11 (78.6%) | 24 (77.4%) | Ref. |  |
| CT | 4 (36.4%) | 0 (0%) | 0.00 (0.00) | 0.999 |  | 3 (21.4%) | 4 (12.9%) | 0.61 (0.12-3.29) | 0.561 |
| TT | 1 (9.1%) | 1 (6.7%) | 0.43 (0.02-8.04) | 0.571 |  | 0 (0%) | 3 (9.7%) | - | - |
| ***TMPRSS2*** |  |  |  |  |  |  |  |  |  |
| AA | 3 (27.3%) | 4 (26.7%) | Ref. |  |  | 3 (21.4%) | 14 (45.2%) | Ref. |  |
| AG | 8(72.7%) | 9 (60%) | 0.84 (0.14-4.97) | 0.851 |  | 5 (35.7%9 | 13 (41.9%) | 0.56 (0.11-2.81) | 0.479 |
| GG | 0 (0%) | 2 (13.3%) | - | - |  | 6 (42.9%) | 4 (12.9%) | 0.14 (0.02-0.84) | **0.032** |

**Table S4.** Allele combination analysis

| **Allele combination analysis with response (n=330, adjusted by sex)** | | | | | | | | |  |
| --- | --- | --- | --- | --- | --- | --- | --- | --- | --- |
| ***ACE2***  **(rs2285666)** | ***MX1***  **(rs469390)** | | ***TMPRSS2***  **(rs2070788)** | **Freq** | | OR (95% CI) | | p-value |  |
| T | A | | G | 0.2474 | | 1 | | - |  |
| C | G | | A | 0.2196 | | 0.76 (0.29-2.01) | | 0.58 |  |
| T | G | | A | 0.175 | | 1.90 (0.58-6.26) | | 0.29 |  |
| C | A | | G | 0.1316 | | 1.45 (0.30-7.05) | | 0.65 |  |
| T | A | | A | 0.1011 | | 0.25 (0.06- 1.09) | | 0.066 |  |
| C | A | | A | 0.079 | | 6.27 (1.00- 39.22) | | **0.051** |  |
| T | G | | G | 0.0297 | | 0.71 (0.06- 7.98) | | 0.78 |  |
| C | G | | G | 0.0167 | | 3.53 (0.03- 442.24) | | 0.61 |  |
| Global allele combination analysis p-value: **0.42**  **Table S5.** Gene expression for asymptomatic/mild patients vs. severe/critical patients. | | | | | | | | |  |
| **Gene** | | **Asymptomatic/mild disease**  **RQ median (CI 95%9)** | | | **Severe/critical disease**  **RQ median (CI 95%)** | | **U de Mann-Whitney**  **p-value** | | |
| ***ERG*** | | 0.020 (0.020-0.030) | | | 0.030 (0.020-0.030) | | 0.160 | | |
| ***ETV5*** | | 0.970 (0.880-1.110) | | | 0.900 (0.760-1.020) | | 0.283 | | |
| ***AR*** | | 0.840 (0.770-0.990) | | | 0.700 (0.520-0.840) | | **0.002** | | |
| ***MX1*** | | 0.945 (0.780-1.080) | | | 0.790 (0.580-0.960) | | **0.036** | | |
| ***ACE2*** | | 0.644 (0.582-0.742) | | | 0.697 (0.558-0.834) | | 0.969 | | |
| ***TMPRSS2*** | | 0.619 (0.511-0.744) | | | 0.649 (0.530-0.866) | | 0.646 | | |

In bold statistically significant values.

**Table S6.** Variants details, changes in transcripts and malignancy calculated with VEP (Variant Effect Predictor).

| **Gene** | **rs_ID** | **Chr** | **Position** | **Location relative to gene** | **EA^*^** | **EAF^*^** | **Transcript Feature** | **Biotype** |
| --- | --- | --- | --- | --- | --- | --- | --- | --- |
| *ACE2* | rs2285666 | X | 15592225 | splice donor region variant, intron variant | T/G/A | 0.35 | [ENST00000252519.8](https://www.ensembl.org/Homo_sapiens/Transcript/Summary?db=core;t=ENST00000252519.8;tl=Ifliwatv4vIlUn0h-8424140), [ENST00000427411.2](https://www.ensembl.org/Homo_sapiens/Transcript/Summary?db=core;t=ENST00000427411.2;tl=Ifliwatv4vIlUn0h-8424140), [ENST00000678046.1](https://www.ensembl.org/Homo_sapiens/Transcript/Summary?db=core;t=ENST00000678046.1;tl=Ifliwatv4vIlUn0h-8424140), [ENST00000678073.1](https://www.ensembl.org/Homo_sapiens/Transcript/Summary?db=core;t=ENST00000678073.1;tl=Ifliwatv4vIlUn0h-8424140), [ENST00000679212.1](https://www.ensembl.org/Homo_sapiens/Transcript/Summary?db=core;t=ENST00000679212.1;tl=Ifliwatv4vIlUn0h-8424140), [ENST00000679278.1](https://www.ensembl.org/Homo_sapiens/Transcript/Summary?db=core;t=ENST00000679278.1;tl=Ifliwatv4vIlUn0h-8424140), [ENST00000680121.1](https://www.ensembl.org/Homo_sapiens/Transcript/Summary?db=core;t=ENST00000680121.1;tl=Ifliwatv4vIlUn0h-8424140) | protein coding |
|  |  |  |  | splice donor region variant, intron variant, NMD transcript variant |  |  | [ENST00000649243.1](https://www.ensembl.org/Homo_sapiens/Transcript/Summary?db=core;t=ENST00000649243.1;tl=Ifliwatv4vIlUn0h-8424140), [ENST00000679162.1](https://www.ensembl.org/Homo_sapiens/Transcript/Summary?db=core;t=ENST00000679162.1;tl=Ifliwatv4vIlUn0h-8424140) | nonsense mediated decay |
| *MX1* | rs469390 | 21 | 41446003 | missense variant | A | 0.462 | [ENST00000288383.11](https://www.ensembl.org/Homo_sapiens/Transcript/Summary?db=core;t=ENST00000288383.11;tl=dfE7UhbBQzoOPrWB-8424143), [ENST00000398598.8](https://www.ensembl.org/Homo_sapiens/Transcript/Summary?db=core;t=ENST00000398598.8;tl=dfE7UhbBQzoOPrWB-8424143), [ENST00000398600.6](https://www.ensembl.org/Homo_sapiens/Transcript/Summary?db=core;t=ENST00000398600.6;tl=dfE7UhbBQzoOPrWB-8424143), [ENST00000413778.6](https://www.ensembl.org/Homo_sapiens/Transcript/Summary?db=core;t=ENST00000413778.6;tl=dfE7UhbBQzoOPrWB-8424143), [ENST00000417963.6](https://www.ensembl.org/Homo_sapiens/Transcript/Summary?db=core;t=ENST00000417963.6;tl=dfE7UhbBQzoOPrWB-8424143), [ENST00000419044.6](https://www.ensembl.org/Homo_sapiens/Transcript/Summary?db=core;t=ENST00000419044.6;tl=dfE7UhbBQzoOPrWB-8424143), [ENST00000424365.6](https://www.ensembl.org/Homo_sapiens/Transcript/Summary?db=core;t=ENST00000424365.6;tl=dfE7UhbBQzoOPrWB-8424143), [ENST00000455164.6](https://www.ensembl.org/Homo_sapiens/Transcript/Summary?db=core;t=ENST00000455164.6;tl=dfE7UhbBQzoOPrWB-8424143), [ENST00000619682.1](https://www.ensembl.org/Homo_sapiens/Transcript/Summary?db=core;t=ENST00000619682.1;tl=dfE7UhbBQzoOPrWB-8424143), [ENST00000679386.1](https://www.ensembl.org/Homo_sapiens/Transcript/Summary?db=core;t=ENST00000679386.1;tl=dfE7UhbBQzoOPrWB-8424143), [ENST00000679408.1](https://www.ensembl.org/Homo_sapiens/Transcript/Summary?db=core;t=ENST00000679408.1;tl=dfE7UhbBQzoOPrWB-8424143), [ENST00000679445.1](https://www.ensembl.org/Homo_sapiens/Transcript/Summary?db=core;t=ENST00000679445.1;tl=dfE7UhbBQzoOPrWB-8424143), [ENST00000679464.1](https://www.ensembl.org/Homo_sapiens/Transcript/Summary?db=core;t=ENST00000679464.1;tl=dfE7UhbBQzoOPrWB-8424143), [ENST00000679543.1](https://www.ensembl.org/Homo_sapiens/Transcript/Summary?db=core;t=ENST00000679543.1;tl=dfE7UhbBQzoOPrWB-8424143), [ENST00000679626.1](https://www.ensembl.org/Homo_sapiens/Transcript/Summary?db=core;t=ENST00000679626.1;tl=dfE7UhbBQzoOPrWB-8424143), [ENST00000679705.1](https://www.ensembl.org/Homo_sapiens/Transcript/Summary?db=core;t=ENST00000679705.1;tl=dfE7UhbBQzoOPrWB-8424143), [ENST00000679911.1](https://www.ensembl.org/Homo_sapiens/Transcript/Summary?db=core;t=ENST00000679911.1;tl=dfE7UhbBQzoOPrWB-8424143), [ENST00000680176.1](https://www.ensembl.org/Homo_sapiens/Transcript/Summary?db=core;t=ENST00000680176.1;tl=dfE7UhbBQzoOPrWB-8424143), [ENST00000680182.1](https://www.ensembl.org/Homo_sapiens/Transcript/Summary?db=core;t=ENST00000680182.1;tl=dfE7UhbBQzoOPrWB-8424143), [ENST00000680347.1](https://www.ensembl.org/Homo_sapiens/Transcript/Summary?db=core;t=ENST00000680347.1;tl=dfE7UhbBQzoOPrWB-8424143), [ENST00000680364.1](https://www.ensembl.org/Homo_sapiens/Transcript/Summary?db=core;t=ENST00000680364.1;tl=dfE7UhbBQzoOPrWB-8424143), [ENST00000680536.1](https://www.ensembl.org/Homo_sapiens/Transcript/Summary?db=core;t=ENST00000680536.1;tl=dfE7UhbBQzoOPrWB-8424143), [ENST00000680629.1](https://www.ensembl.org/Homo_sapiens/Transcript/Summary?db=core;t=ENST00000680629.1;tl=dfE7UhbBQzoOPrWB-8424143), [ENST00000680760.1](https://www.ensembl.org/Homo_sapiens/Transcript/Summary?db=core;t=ENST00000680760.1;tl=dfE7UhbBQzoOPrWB-8424143), [ENST00000680776.1](https://www.ensembl.org/Homo_sapiens/Transcript/Summary?db=core;t=ENST00000680776.1;tl=dfE7UhbBQzoOPrWB-8424143), [ENST00000680942.1](https://www.ensembl.org/Homo_sapiens/Transcript/Summary?db=core;t=ENST00000680942.1;tl=dfE7UhbBQzoOPrWB-8424143), [ENST00000681039.1](https://www.ensembl.org/Homo_sapiens/Transcript/Summary?db=core;t=ENST00000681039.1;tl=dfE7UhbBQzoOPrWB-8424143), [ENST00000681191.1](https://www.ensembl.org/Homo_sapiens/Transcript/Summary?db=core;t=ENST00000681191.1;tl=dfE7UhbBQzoOPrWB-8424143), [ENST00000681266.1](https://www.ensembl.org/Homo_sapiens/Transcript/Summary?db=core;t=ENST00000681266.1;tl=dfE7UhbBQzoOPrWB-8424143), [ENST00000681415.1](https://www.ensembl.org/Homo_sapiens/Transcript/Summary?db=core;t=ENST00000681415.1;tl=dfE7UhbBQzoOPrWB-8424143), [ENST00000681607.1](https://www.ensembl.org/Homo_sapiens/Transcript/Summary?db=core;t=ENST00000681607.1;tl=dfE7UhbBQzoOPrWB-8424143), [ENST00000681671.1](https://www.ensembl.org/Homo_sapiens/Transcript/Summary?db=core;t=ENST00000681671.1;tl=dfE7UhbBQzoOPrWB-8424143), [ENST00000681849.1](https://www.ensembl.org/Homo_sapiens/Transcript/Summary?db=core;t=ENST00000681849.1;tl=dfE7UhbBQzoOPrWB-8424143), [ENST00000681857.1](https://www.ensembl.org/Homo_sapiens/Transcript/Summary?db=core;t=ENST00000681857.1;tl=dfE7UhbBQzoOPrWB-8424143), [ENST00000681867.1](https://www.ensembl.org/Homo_sapiens/Transcript/Summary?db=core;t=ENST00000681867.1;tl=dfE7UhbBQzoOPrWB-8424143), [ENST00000681896.1](https://www.ensembl.org/Homo_sapiens/Transcript/Summary?db=core;t=ENST00000681896.1;tl=dfE7UhbBQzoOPrWB-8424143) | protein coding |
|  |  |  |  | upstream gene variant |  |  | [ENST00000411427.3](https://www.ensembl.org/Homo_sapiens/Transcript/Summary?db=core;t=ENST00000411427.3;tl=dfE7UhbBQzoOPrWB-8424143) | lncRNA |
|  |  |  |  |  |  |  | [ENST00000491110.1](https://www.ensembl.org/Homo_sapiens/Transcript/Summary?db=core;t=ENST00000491110.1;tl=dfE7UhbBQzoOPrWB-8424143) | retained intron |
|  |  |  |  | downstream gene variant |  |  | [ENST00000441677.6](https://www.ensembl.org/Homo_sapiens/Transcript/Summary?db=core;t=ENST00000441677.6;tl=dfE7UhbBQzoOPrWB-8424143), [ENST00000679528.1](https://www.ensembl.org/Homo_sapiens/Transcript/Summary?db=core;t=ENST00000679528.1;tl=dfE7UhbBQzoOPrWB-8424143) | retained intron |
|  |  |  |  | non coding transcript exon variant |  |  | [ENST00000467510.2](https://www.ensembl.org/Homo_sapiens/Transcript/Summary?db=core;t=ENST00000467510.2;tl=dfE7UhbBQzoOPrWB-8424143), [ENST00000486275.2](https://www.ensembl.org/Homo_sapiens/Transcript/Summary?db=core;t=ENST00000486275.2;tl=dfE7UhbBQzoOPrWB-8424143), [ENST00000680637.1](https://www.ensembl.org/Homo_sapiens/Transcript/Summary?db=core;t=ENST00000680637.1;tl=dfE7UhbBQzoOPrWB-8424143), [ENST00000681382.1](https://www.ensembl.org/Homo_sapiens/Transcript/Summary?db=core;t=ENST00000681382.1;tl=dfE7UhbBQzoOPrWB-8424143) | retained intron |
|  |  |  |  | missense variant, NMD transcript variant |  |  | [ENST00000681944.1](https://www.ensembl.org/Homo_sapiens/Transcript/Summary?db=core;t=ENST00000681944.1;tl=dfE7UhbBQzoOPrWB-8424143) | nonsense mediated decay |
| *TMPRSS2* | rs2070788 | 21 | 41470061 | intron variant | A | 0.603 | [ENST00000332149.10](https://www.ensembl.org/Homo_sapiens/Transcript/Summary?db=core;t=ENST00000332149.10;tl=30hP1IFMFxxR2eK0-8424141), [ENST00000398585.7](https://www.ensembl.org/Homo_sapiens/Transcript/Summary?db=core;t=ENST00000398585.7;tl=30hP1IFMFxxR2eK0-8424141), [ENST00000424093.6](https://www.ensembl.org/Homo_sapiens/Transcript/Summary?db=core;t=ENST00000424093.6;tl=30hP1IFMFxxR2eK0-8424141), [ENST00000454499.6](https://www.ensembl.org/Homo_sapiens/Transcript/Summary?db=core;t=ENST00000454499.6;tl=30hP1IFMFxxR2eK0-8424141), [ENST00000458356.6](https://www.ensembl.org/Homo_sapiens/Transcript/Summary?db=core;t=ENST00000458356.6;tl=30hP1IFMFxxR2eK0-8424141), [ENST00000676973.1](https://www.ensembl.org/Homo_sapiens/Transcript/Summary?db=core;t=ENST00000676973.1;tl=30hP1IFMFxxR2eK0-8424141), [ENST00000678171.1](https://www.ensembl.org/Homo_sapiens/Transcript/Summary?db=core;t=ENST00000678171.1;tl=30hP1IFMFxxR2eK0-8424141), [ENST00000678348.1](https://www.ensembl.org/Homo_sapiens/Transcript/Summary?db=core;t=ENST00000678348.1;tl=30hP1IFMFxxR2eK0-8424141), [ENST00000678743.1](https://www.ensembl.org/Homo_sapiens/Transcript/Summary?db=core;t=ENST00000678743.1;tl=30hP1IFMFxxR2eK0-8424141), [ENST00000679016.1](https://www.ensembl.org/Homo_sapiens/Transcript/Summary?db=core;t=ENST00000679016.1;tl=30hP1IFMFxxR2eK0-8424141), [ENST00000679054.1](https://www.ensembl.org/Homo_sapiens/Transcript/Summary?db=core;t=ENST00000679054.1;tl=30hP1IFMFxxR2eK0-8424141), [ENST00000679263.1](https://www.ensembl.org/Homo_sapiens/Transcript/Summary?db=core;t=ENST00000679263.1;tl=30hP1IFMFxxR2eK0-8424141) | protein coding |
|  |  |  |  | upstream gene variant |  |  | [ENST00000469395.1](https://www.ensembl.org/Homo_sapiens/Transcript/Summary?db=core;t=ENST00000469395.1;tl=30hP1IFMFxxR2eK0-8424141) | retained intron |
|  |  |  |  | intron variant, NMD transcript variant |  |  | [ENST00000677680.1](https://www.ensembl.org/Homo_sapiens/Transcript/Summary?db=core;t=ENST00000677680.1;tl=30hP1IFMFxxR2eK0-8424141), [ENST00000678959.1](https://www.ensembl.org/Homo_sapiens/Transcript/Summary?db=core;t=ENST00000678959.1;tl=30hP1IFMFxxR2eK0-8424141), [ENST00000679181.1](https://www.ensembl.org/Homo_sapiens/Transcript/Summary?db=core;t=ENST00000679181.1;tl=30hP1IFMFxxR2eK0-8424141) | nonsense mediated decay |
|  |  |  |  | intron variant, non coding transcript variant |  |  | [ENST00000678617.1](https://www.ensembl.org/Homo_sapiens/Transcript/Summary?db=core;t=ENST00000678617.1;tl=30hP1IFMFxxR2eK0-8424141) | processed transcript |
|  |  |  |  | 3’ UTR variant |  |  | [ENST00000679386.1](https://www.ensembl.org/Homo_sapiens/Transcript/Summary?db=core;t=ENST00000679386.1;tl=30hP1IFMFxxR2eK0-8424141) | protein coding |

EA: Effect allele; EAF: Effect allele frequency; NMD: Nonsense Mediated Decay.

| **Table S7.** Primers genotyping details.   | **Gene** | **SNP ID** | **Assay ID** | **Sequence** | | --- | --- | --- | --- | | *ACE2* | rs2285666 | C___2551626_1_ | ATAATCACTACTAAAAATTAGTAGC**[C/T]**TACCTGGTTCAAGTAATAAGCATTC | | *MX1* | rs469390 | C___2274997_10 | CTGATGTTTTTCTTCTTGACAGAAA**[A/G]**TTAATGCCTTTAATCAGGACATCAC | | *TMPRSS2* | rs2070788 | C___2592038_1_ | GATTTGTTGTCTGTATGGCCTAGAC**[A/G]**CTTTTGAGAAGGATATAAACAATAT |   ***Table S8. Polymorphism frequencies and Hardy-Weinberg equilibrium.***   |  |  | **Iberian population in Spain**  **Proportion** | **Our study population Proportion (count)** | **Hardy-Weinberg equilibrium, N=330**  **(p -value)** | | --- | --- | --- | --- | --- | | *MX1* (rs469390) | A allele | 0.645 | 0.56 (367) | 0.74 | | G allele | 0.355 | 0.44 (293) | | A/A genotype | 0.449 | 0.3 (100) | | A/G genotype | 0.393 | 0.51 (167) | | G/G genotype | 0.159 | 0.19 (63) | | *ACE2* (rs2285666) | C allele | 0.750 | 0.81 (534) | **<0.0001**** | | T allele | 0.250 | 0.19 (126) | | C/C genotype | 0.262 | 0.72 (239) | | C/T genotype | 0.206 | 0.17 (56) | | T/T genotype | 0.028 | 0.11 (35) | | *TMPRSS2* (rs2070788) | A allele | 0.500 | 0.58 (379) | 0.73 | | G allele | 0.500 | 0.42 (279) | | A/A genotype | 0.252 | 0.34 (111) | | A/G genotype | 0.495 | 0.48 (157) | | G/G genotype | 0.252 | 0.19 (61) |   ***ACE2* rs2285666 is located in X chromosome.  **Table S9. Details of primers mRNAs probes and TaqMan gene expression assays**   | .**Gene** | **Primer mRNA sequence** | | --- | --- | | *ERG* | Forward: 5’-ATGGACAGACTTCCAAGAT-3’  Reverse: 5’-TTCATCAGGAGAGTTCCTT-3’ | | *ETV5* | Forward: TTTATGGTCCCAGGGAAAT  Reverse: GAGCCAGATCTGTGTCCAA | | *AR* | Forward: GACATGCGTTTGGAGACTGC  Reverse: TTCCCTTCAGCGGCTCTTTT | | *MX1* | Forward: GAGGCAAGGTCAGTTACCAGG  Reverse: CTGATTCCCATTCCTTCCCCG | | **Gene** | **Assay ID** | | *ACE2* | Hs01085333_m1 | | *TMPRSS2* | Hs01122322_m1 |   Supplementary Material 1. Meta-analysis  Present meta-analysis was performed using the three selected SNPs, located in *ACE2* (rs2285666), *TMPRSS2* (rs2070788) and *MX1* (rs469390) genes. The search was developed by using several websites such as [**https://pubmed.ncbi.nlm.nih.gov/**](https://pubmed.ncbi.nlm.nih.gov/), [**https://www.snpedia.com/**](https://www.snpedia.com/), and [**https://www.ebi.ac.uk/**](https://www.ebi.ac.uk/). After searching, studies referred to these SNPs were filtered following two steps for their inclusion: a) At least one of the SNPs was related to COVID-19; b) case-control studies, or severity-based studies. We excluded reports with no data of allelic frequencies in the study. No case-control or cases studies were found for rs469390 (*MX1*). Finally, 12 published articles were included in the meta-analysis (2 studies related to polymorphism in *TMPRSS2* gene (rs207088) and 10 articles providing information about rs2285666, in *ACE2* gene), and our own results were added to those in previous publications.  To carry out the meta-analysis, we used MetaGenyo platform: <http://bioinfo.genyo.es/metagenyo/> (1) which reports statistical data among different genetic variants and presence and severity of COVID-19. A total of 12 studies about rs2285666 (*ACE2*) and rs2070788 (*TMPRSS2*) were added to our results and included in the meta-analysis. No previous studies related to rs469390 (*MX1*) were found, although it has been suggested as a lung infection susceptibility marker increasing *TMPRSS2* expression in COVID-19 (2). From all these 12 articles, 10 provided information about rs2285666 (all of them comparing COVID-19 severity) and 2 about rs2070788 (based on disease severity). Three of the studies involving rs2285666 (3-5) did not adapt to HWE (*p* < 0.05), but they were included in the meta-analysis because this assumption of equilibrium was checked in original articles. See details in Table S9.  Table S9. Summary of the results obtained in present meta-analysis.   | **COVID-19 severity studies** | | | | | | | --- | --- | --- | --- | --- | --- | | **SNP** | **Studies/**  **Publications** | **HWE** | **Significance** | | **OR**  **[95% CI]** | | **rs2285666**  (*ACE2*) | (Iran) (6) | 0.8023 | Codominant Model | 0.514 | 0.838  [0.40;1.43] | | (Egypt) (7) | 0.9618 | | (Polish) (8) | 0.9419 |  |  |  | | (Spain) (9) | 0.9618 |  |  |  | | (Germany) (3) | 0 | Recessive Model | 0.933 | 1.027  [0.54; 1.94] | | (Turkey) (5) | 0 | | (Iran) (10) | 0.9419 |  |  |  | | (Mexico) (4) | 0 |  |  |  | | (Iran) (11) | 0.4797 | Dominant Model | 0.455 | 0.789  [0.42; 1.47] | | (Spain) (12) | 0.9618 | | Our study (Spain) | 0.9618 |  |  |  | | **rs2070788**  (*TMPRSS2*) | (Netherlands) (13) | | 0.9945 | | --- | | Codominant Model | 0.101 | 1.196  [0.96; 1.48] | | (Germany) (14) | 0.8703 | Recessive Model | 0.439 | 1.160  [0.80; 1.69] | | Our study (Spain) | 0.9945 | Dominant Model | 0.070 | 1.359  [0.97; 1.90] |   HWE: Hardy-Weinberg Equilibrium, OR: Odds Ratio, 95% CI: Confidence Interval.  No relationship was found between these SNPs and the severity of COVID-19, although a trend was observed using a dominant model for rs2070788 in meta-analysis to disease severity (*p* = 0.070; Table S9). We are conscious of the sample limitation in present meta-analysis, a wider cohort will improve present statistical data for COVID-19 severity.  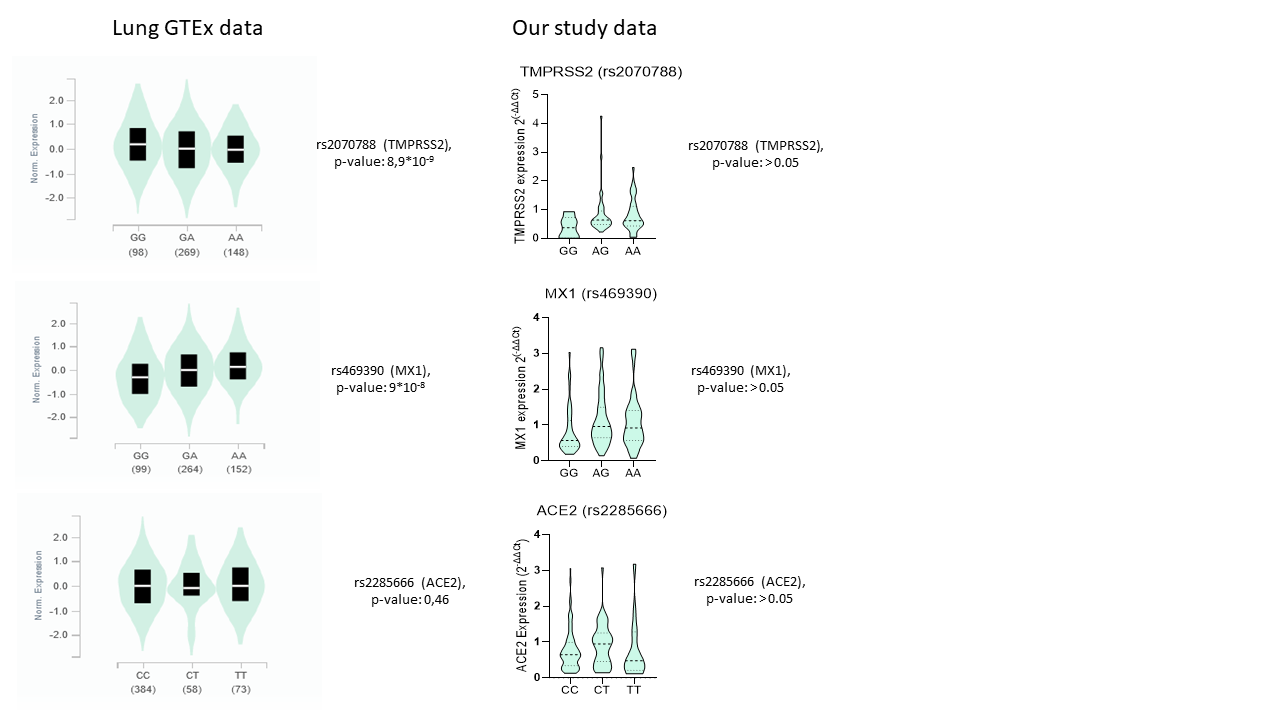  **Figure S1**. Expression data using GTEx online database in lung in comparison with our serum expression data of rs2070788 (*TMPRSS2*), rs469390 (*MX1*) and rs2285666 (*ACE2*). |
| --- | --- | --- | --- | --- | --- | --- | --- | --- | --- | --- | --- | --- | --- | --- | --- | --- | --- | --- | --- | --- | --- | --- | --- | --- | --- | --- | --- | --- | --- | --- | --- | --- | --- | --- | --- | --- | --- | --- | --- | --- | --- | --- | --- | --- | --- | --- | --- | --- | --- | --- | --- | --- | --- | --- | --- | --- | --- | --- | --- | --- | --- | --- | --- | --- | --- | --- | --- | --- | --- | --- | --- | --- | --- | --- | --- | --- | --- | --- | --- | --- | --- | --- | --- | --- | --- | --- | --- | --- | --- | --- | --- | --- | --- | --- | --- | --- | --- | --- | --- | --- | --- | --- | --- | --- | --- | --- | --- | --- | --- | --- | --- | --- | --- | --- | --- | --- | --- | --- | --- | --- | --- | --- | --- | --- | --- | --- | --- | --- | --- | --- | --- | --- | --- | --- | --- | --- | --- | --- | --- | --- | --- | --- | --- | --- | --- | --- | --- | --- | --- | --- | --- | --- | --- | --- | --- | --- | --- | --- | --- | --- | --- | --- | --- | --- |


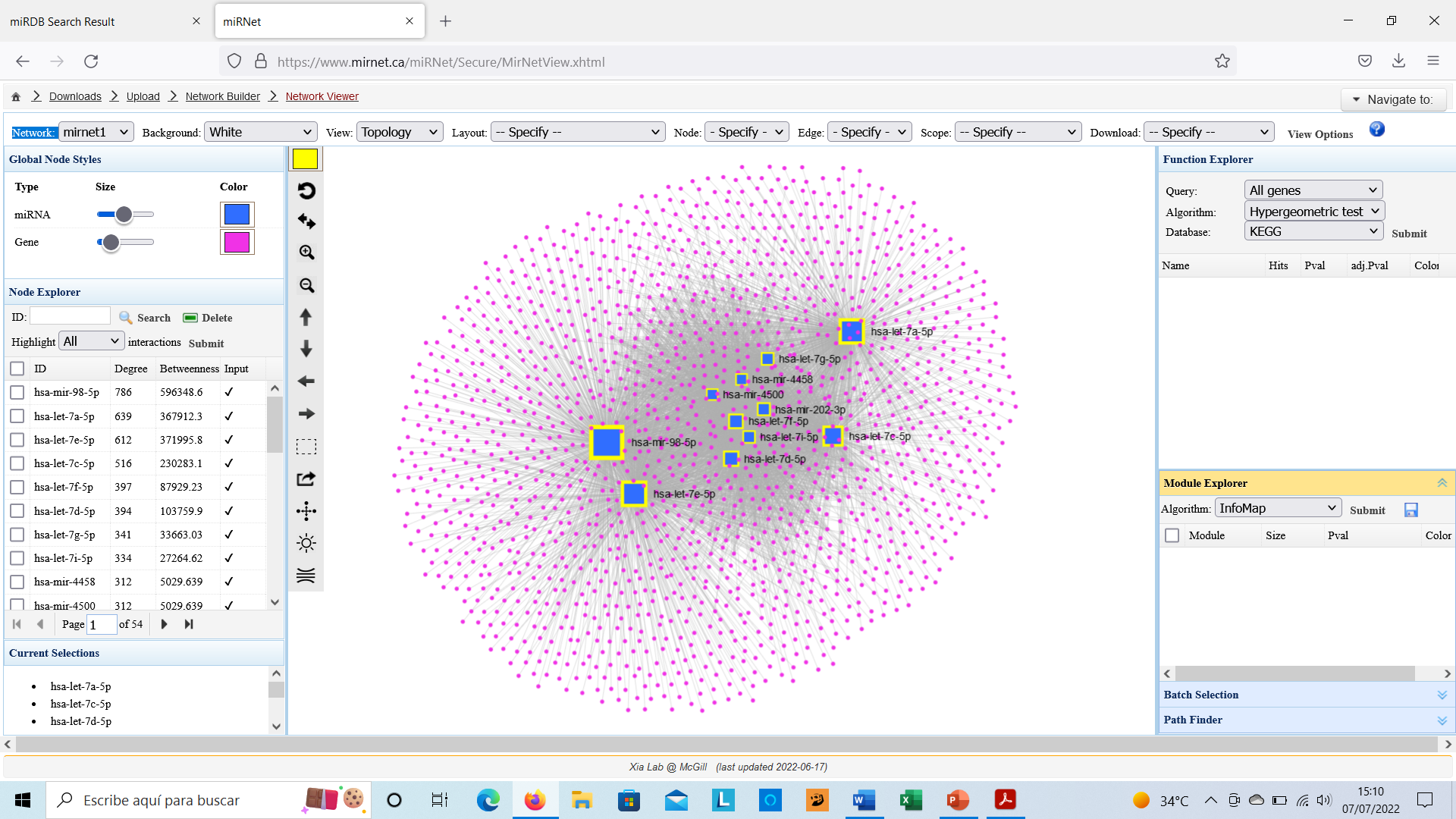


**Figure S2**. The miRNA-mRNA network of selected miRNAs.  The microRNAs hsa-miR-98-5p, hsa-let-7e-5p, hsa-let-7a-5p, hsa-let-7c-5p, hsa-let-7d-5p, hsa-let-7f-5p, hsa-miR-202-3p, hsa-let-7g-5p, hsa-let-7i-5p, hsa-miR-4458 & hsa-miR-4500 were highlighted by the miRNet network as master regulators of the genes studied in this work.


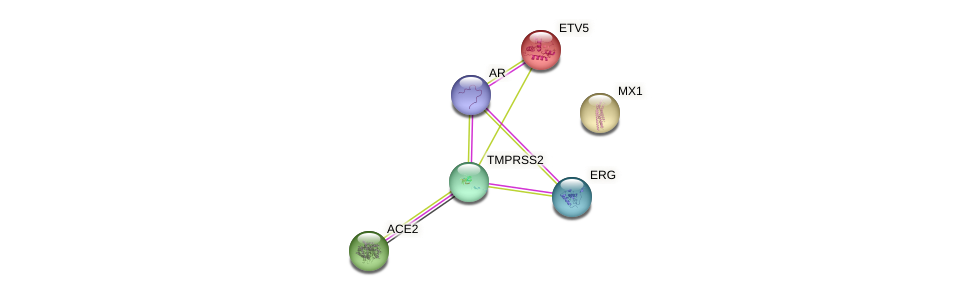


**Figure S3**. STRING protein-protein network with genes of present study.

**Supplementary References**

(1) Martorell-Marugan J, Toro-Dominguez D, Alarcon-Riquelme ME, Carmona-Saez P. MetaGenyo: a web tool for meta-analysis of genetic association studies. BMC Bioinformatics 2017 -12-16;18(1):563.

(2) Irham LM, Chou W, Calkins MJ, Adikusuma W, Hsieh S, Chang W. Genetic variants that influence SARS-CoV-2 receptor *TMPRSS2* expression among population cohorts from multiple continents. Biochem Biophys Res Commun 2020 -08-20;529(2):263-269.

(3) Möhlendick B, Schönfelder K, Breuckmann K, Elsner C, Babel N, Balfanz P, *et al*. *ACE2* polymorphism and susceptibility for SARS-CoV-2 infection and severity of COVID-19. Pharmacogenet Genomics 2021 -10-01;31(8):165-171.

(4) Martínez-Gómez LE, Herrera-López B, Martinez-Armenta C, Ortega-Peña S, Camacho-Rea MDC, Suarez-Ahedo C, *et al*. ACE and *ACE2* Gene Variants Are Associated With Severe Outcomes of COVID-19 in Men. Front Immunol 2022;13:812940.

(5) Karakaş Çelik S, Çakmak Genç G, Pişkin N, Açikgöz B, Altinsoy B, Kurucu İşsiz B, *et al*. Polymorphisms of ACE (I/D) and *ACE2* receptor gene (Rs2106809, Rs2285666) are not related to the clinical course of COVID-19: A case study. J Med Virol 2021 -10;93(10):5947-5952.

(6) Alimoradi N, Sharqi M, Firouzabadi D, Sadeghi MM, Moezzi MI, Firouzabadi N. SNPs of ACE1 (rs4343) and *ACE2* (rs2285666) genes are linked to SARS-CoV-2 infection but not with the severity of disease. Virol J 2022 -03-19;19(1):48.

(7) Abdelsattar S, Kasemy ZA, Ewida SF, Abo-Elsoud RaA, Zytoon AA, Abdelaal GA, *et al*. *ACE2* and *TMPRSS2* SNPs as Determinants of Susceptibility to, and Severity of, a COVID-19 Infection. Br J Biomed Sci 2022;79:10238.

(8) Sienko J, Marczak I, Kotowski M, Bogacz A, Tejchman K, Sienko M, *et al*. Association of *ACE2* Gene Variants with the Severity of COVID-19 Disease-A Prospective Observational Study. Int J Environ Res Public Health 2022 -10-02;19(19).

(9) Khalilzadeh F, Sakhaee F, Sotoodehnejadnematalahi F, Zamani MS, Ahmadi I, Anvari E, *et al*. Angiotensin-converting enzyme 2 rs2285666 polymorphism and clinical parameters as the determinants of COVID-19 severity in Iranian population. Int J Immunogenet 2022 -10;49(5):325-332.

(10) Jevnikar K, Lapajne L, Petrovič D, Meglič A, Logar M, Vidovič Valentinčič N, *et al*. The Role of ACE, *ACE2*, and AGTR2 Polymorphisms in COVID-19 Severity and the Presence of COVID-19-Related Retinopathy. Genes (Basel) 2022 -06-21;13(7).

(11) Najafi M, Mahdavi MR. Association investigations between ACE1 and *ACE2* polymorphisms and severity of COVID-19 disease. Mol Genet Genomics 2022 -10-18.

(12) Sabater Molina M, Nicolás Rocamora E, Bendicho AI, Vázquez EG, Zorio E, Rodriguez FD, *et al*. Polymorphisms in ACE, *ACE2*, AGTR1 genes and severity of COVID-19 disease. PLoS One 2022;17(2):e0263140.

(13) Akin S, Schriek P, van Nieuwkoop C, Neuman RI, Meynaar I, van Helden EJ, *et al*. A low aldosterone/renin ratio and high soluble *ACE2* associate with COVID-19 severity. J Hypertens 2022 -03-01;40(3):606-614.

(14) Schönfelder K, Breuckmann K, Elsner C, Dittmer U, Fistera D, Herbstreit F, *et al*. Transmembrane serine protease 2 Polymorphisms and Susceptibility to Severe Acute Respiratory Syndrome Coronavirus Type 2 Infection: A German Case-Control Study. Front Genet 2021;12:667231.
